# The global RNA–RNA interactome of *Klebsiella pneumoniae* unveils a small RNA regulator of cell division

**DOI:** 10.1073/pnas.2317322121

**Published:** 2024-02-20

**Authors:** Eric Ruhland, Malte Siemers, Ruman Gerst, Felix Späth, Laura Nicole Vogt, Marc Thilo Figge, Kai Papenfort, Kathrin Sophie Fröhlich

**Affiliations:** ^a^Institute of Microbiology, Faculty of Biological Sciences, Friedrich Schiller University, Jena 07743, Germany; ^b^Cluster of Excellence Balance of the Microverse, Friedrich Schiller University, Jena 07743, Germany; ^c^Faculty of Biological Sciences, Friedrich Schiller University, Jena 07743, Germany; ^d^Applied Systems Biology, Leibniz Institute for Natural Product Research and Infection Biology–Hans Knöll Institute, Jena 07745, Germany

**Keywords:** *Klebsiella pneumoniae*, Hfq, RIL-seq, small RNA, SOS response

## Abstract

The ubiquitous RNA chaperone Hfq is involved in the regulation of key biological processes in many species across the bacterial kingdom. In the opportunistic human pathogen *Klebsiella pneumoniae*, deletion of the *hfq* gene affects the global transcriptome, virulence, and stress resistance; however, the ligands of the major RNA-binding protein in this species have remained elusive. In this study, we have combined transcriptomic, co-immunoprecipitation, and global RNA interactome analyses to compile an inventory of conserved and species-specific RNAs bound by Hfq and to monitor Hfq-mediated RNA–RNA interactions. In addition to dozens of RNA–RNA pairs, our study revealed an Hfq-dependent small regulatory RNA (sRNA), DinR, which is processed from the 3′ terminal portion of *dinI* mRNA. Transcription of *dinI* is controlled by the master regulator of the SOS response, LexA. As DinR accumulates in *K. pneumoniae* in response to DNA damage, the sRNA represses translation of the *ftsZ* transcript by occupation of the ribosome binding site. Ectopic overexpression of DinR causes depletion of *ftsZ* mRNA and inhibition of cell division, while deletion of *dinR* antagonizes cell elongation in the presence of DNA damage. Collectively, our work highlights the important role of RNA-based gene regulation in *K. pneumoniae* and uncovers the central role of DinR in LexA-controlled division inhibition during the SOS response.

## Significance

Albeit it has long been known that the RNA chaperone Hfq is critical for *Klebsiella pneumoniae* virulence and adaptation to various environmental stress conditions, the landscape of Hfq-associated transcripts in this organism has not been explored. In a global search for RNA–RNA interactions, we annotated targets of conserved as well as previously unknown regulatory RNAs. We show that a *Klebsiella*-specific sRNA (small regulatory RNA), termed DinR, contributes to inhibition of cell cycle progression in the response to DNA damage by interfering with translation of the essential divisome component FtsZ. Acting at the posttranscriptional level, DinR complements other conserved mechanisms of FtsZ deactivation in the SOS response and fosters filamentation of *K. pneumoniae*.

*Klebsiella pneumoniae* is a ubiquitous member of the gram-negative Enterobacteriaceae. Equipped with a thick polysaccharide capsule that facilitates host immune system evasion, *K. pneumoniae* causes a wide range of severe diseases in humans including urinary and respiratory tract infections, bacteremia, and pyogenic liver abscesses (1, 2). Classical *K. pneumoniae* strains are common nosocomial pathogens typically responsible for infections in immunocompromised patients (3). However, the ability of this bacterium to efficiently accumulate genetic traits that confer resistance toward an increasing number of antibiotics has prompted the World Health Organization to list *K. pneumoniae* as a pathogen of highest priority regarding the development of alternative therapeutic compounds (4).

Bacteria acquire genome-encoded drug resistances either through horizontal gene transfer or by vertical evolution, i.e., the emergence and transmission of de novo mutations which enhance antibiotic tolerance (5). The frequency of mutations in bacteria increases in the presence of active stress responses, like the general stress response, the cellular responses to starvation, the presence of oxidative stress, or membrane damage which all may generate single-stranded DNA (ssDNA) at stalled replication forks and DNA lesions (6, 7). DNA damage typically activates the SOS response, a global regulatory pathway promoting DNA repair while the cell cycle is arrested (8). The two master regulators of the SOS response are the ssDNA-binding protein, RecA, and the transcriptional repressor protein, LexA. Recruitment of RecA to DNA lesions results in the formation of a nucleofilament on ssDNA which stimulates LexA autoproteolysis (9). When cleaved, LexA dissociates from its DNA operator sites—the so-called LexA boxes (10, 11)—to enable expression of ~40 genes in *Escherichia coli* required for DNA repair, DNA damage tolerance, and cell cycle control (10, 12). In *E. coli*, the SOS response stalls cell division to permit time for chromosome repair by activation of SulA which interferes with FtsZ polymerization to inhibit Z-ring formation at midcell (13, 14).

The coordination of the SOS response with additional environmental stresses requires precise fine-tuning of gene expression programs. Gene expression control in bacteria was long considered to occur predominantly at the level of transcription; however, extensive research over the past two decades has uncovered the broad contribution of posttranscriptional regulation involving small, regulatory RNAs (sRNAs) and RNA-binding proteins (RBPs). The majority of bacterial sRNAs act by regulation of trans-encoded messenger RNAs (mRNAs) through short, often imperfect base-pairing interactions (15, 16). The formation of sRNA–mRNA hybrids is generally aided by RBPs, the most prominent being Hfq which can simultaneously bind to an sRNA and its potential target and, given sufficient complementarity, facilitate complex formation (17).

Bacterial sRNAs may repress or stimulate expression of a targeted transcript. In most cases, formation of the RNA duplex occludes the ribosome recognition site of the target. The inhibition of translation initiation increases the susceptibility of the mRNA to ribonucleolytic cleavage and results in rapid decay of the targeted transcript alone or together with the bound sRNA (18, 19).

While hundreds of genomes from clinical *K. pneumoniae* isolates have been sequenced to determine phenotypes relevant to pathogenesis of individual strains (20–22), significantly less is known about the RNA landscape of this bacterium. Importantly, mutation of *hfq* results in deregulation of ~20% of all *K. pneumoniae* genes and has been associated with impaired stress responses and defects in intestinal colonization and systemic infection of mice (23). These results indicate that Hfq-mediated gene expression control is key for various aspects of *K. pneumoniae*’s lifestyle. The close genetic relationship to the genera *Escherichia* and *Salmonella* within the family of Enterobacteriaceae has fostered the extrapolation of results obtained for these two well-studied model organisms of bacterial RNA biology to *Klebsiella*. Indeed, transcriptome analyses have uncovered the conservation of ~50 Hfq-dependent sRNAs in the well-studied clinical isolate MGH 78578, including the highly expressed GcvB, CyaR, and GlmZ (23–25). However, no individual sRNA has been characterized in *Klebsiella* to date, and the strain-specific ligands of Hfq and their regulatory roles are still undetermined.

To address this paucity, we have applied differential RNA sequencing (dRNA-seq) to annotate ~3,750 transcriptional start sites (TSSs) at low and high cell density in the multidrug-resistant *K. pneumoniae* isolate MGH 78578. RNA co-immunoprecipitation and sequencing (RIP-seq) of Hfq-associated RNAs revealed growth phase-dependent and strain-specific transcripts interacting with the RBP, including numerous sRNA candidates encoded on core genome and accessory plasmids. In addition, we have employed RIL-seq [RNA interactions by ligation and sequencing (26)] to globally map the Hfq-dependent RNA interactome of *K. pneumoniae* in early stationary growth. Among thousands of RNA–RNA pairs, we identified previously uncharacterized targets of conserved sRNAs as well as strain-specific interactions. One of the candidate sRNAs, termed DinR, is released from the 3′ end of the LexA-controlled *dinI* mRNA through endonucleolytic cleavage. In the presence of Hfq, DinR recognizes the translation initiation site of *ftsZ* mRNA, resulting in down-regulation of the target and cell filamentation. We therefore propose DinR as the noncoding tier of cell cycle control of the SOS response in *K. pneumoniae*.

## Results

### The Primary Transcriptome of *K. pneumoniae* MGH 78578.

To map the transcriptomes of *Klebsiella* in exponential and stationary growth phase, we determined TSSs by dRNA-seq, a protocol employing the enrichment of primary transcripts through selective degradation of processed species from the total RNA pool (27). We cultivated *K. pneumoniae* MGH 78578, a multidrug-resistant strain isolated from the sputum of a pneumonia patient in 1994 (28), under standard laboratory conditions in rich LB medium at 37 °C and isolated total RNA from samples harvested at two different time points over growth [OD_600_ of 0.25 (mid-exponential phase, MEP) and 2.0 (early stationary phase, ESP), respectively]. Library preparation and deep sequencing yielded at least 7 million cDNA reads per condition mapping to the 5,694,894 bp MGH 78578 genome, covering the main chromosome and five accessory plasmids (pKPN3-7). Individual TSSs were classified regarding their genomic location relative to the annotated coding sequences (CDSs) as proximal (closest TSS of a gene), distal (alternative TSS, upstream of a proximal TSS), internal (within an open reading frame), antisense (expressed from the complementary strand of an annotated gene), or orphan TSSs (no clear association to a flanking gene), respectively (*SI Appendix*, Fig. S1*A*).

In total, we identified 3,748 individual TSSs under the tested conditions (*SI Appendix*, Fig. S1*B* and Dataset S1) and observed an enrichment for 5′UTRs with a length of 20 to 50 nt (*SI Appendix*, Fig. S1*C*). We detected at least two alternative TSSs for 178 genes, and 912 transcripts were differentially regulated between the two examined growth phases (*SI Appendix*, Fig. S1 *B* and *D*). Comparison of our data to a published transcriptome study of strain MGH 78578 cultivated in minimal medium (25) revealed an overlap of more than 1,000 TSSs with our annotation (*SI Appendix*, Fig. S1*E*), confirming the accuracy of our experimental approach.

### The Hfq Interactome in *K. pneumoniae*.

**Fig. 1.**
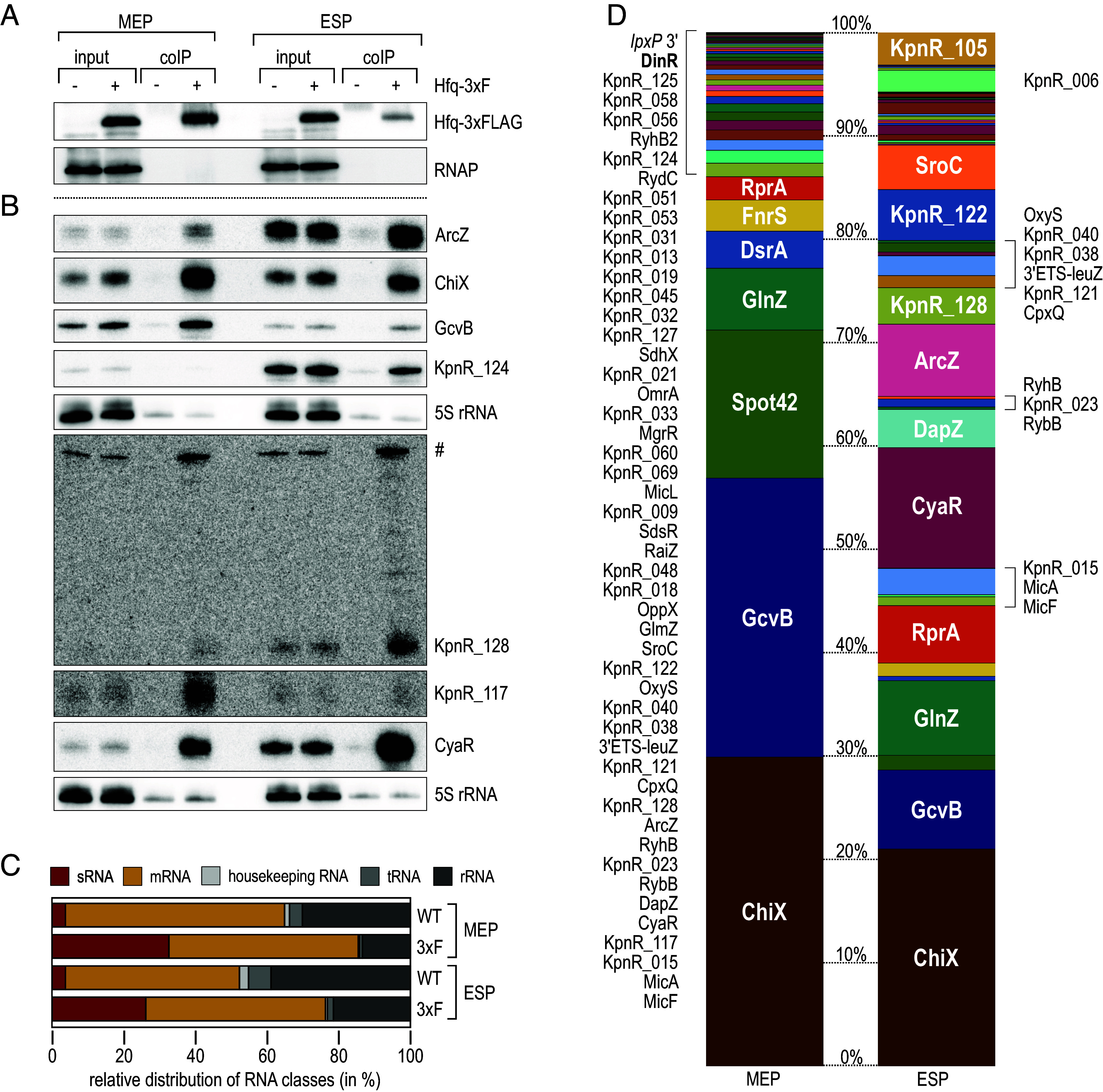
RIP-seq analysis uncovers Hfq-associated sRNAs in *K. pneumoniae*. (*A* and *B*) *K. pneumoniae* MGH 78578 expressing FLAG-tagged Hfq (3xF; +) from the native chromosomal locus were cultivated in LB to MEP (OD_600_ of 0.25) or ESP (OD_600_ of 2.0) and subjected to immunoprecipitation with an anti-FLAG antibody. An untagged WT strain (−) served as control. Protein and RNA samples were collected prior to (input) and after purification (coIP). (*A*) Protein samples were analyzed on western blots to confirm expression and enrichment of the Hfq-3xFLAG protein. RNAP was probed as loading control. (*B*) RNA samples were analyzed on Northern blots to determine expression and enrichment of indicated sRNAs using gene-specific probes. KpnR_128 is expressed from its own promoter internal to an upstream gene. Both transcripts share the same termination site and the ~300 nt upstream transcript (#) is thus detected by the KpnR_128 probe. 5S rRNA served as loading control. (*C*) Relative abundance of different RNA classes recovered from RNA coIPs from WT and cells expressing FLAG-tagged Hfq (3xF) in MEP and ESP. (*D*) Read distribution of sRNAs enriched ≥threefold after coIP with FLAG-tagged Hfq in MEP (*Left*) and ESP (*Right*).

A key role of Hfq in posttranscriptional regulation is to facilitate base-pairing interactions between regulatory sRNAs and their target transcripts. However, the number and identity of specific ligands of the chaperone in *Klebsiella* have remained unknown. To close this gap, we conducted high-throughput sequencing of RNA co-purified with Hfq carrying a C-terminal 3xFLAG epitope (RIP-seq) and included wild-type (WT) MGH 78578 expressing untagged Hfq in our experiment as a control in both MEP and ESP (*SI Appendix*, Fig. S2*A*). We verified that addition of the epitope tag did not impair growth (*SI Appendix*, Fig. S2*B*), and western blot analysis confirmed expression of Hfq-3xFLAG at all phases of growth and specific enrichment of the fusion protein in the RIP-seq protocol (*SI Appendix*, Fig. S2*C* and Fig. 1*A*). Consistently, recovery of the conserved Hfq-dependent sRNAs ChiX, GcvB, CyaR, and ArcZ was strongly enhanced in the Hfq-3xFLAG coIP samples (Fig. 1*B*). When profiling the RNA species co-immunoprecipitating with Hfq in comparison to the control by deep sequencing, we observed an increase in the proportion of sRNAs (8.8-fold in MEP and 7.0-fold in ESP, respectively; Fig. 1*C* and Dataset S2). In addition, 5,277 mRNAs were detected under both conditions, and 450 of them were enriched at least threefold in the coIP fraction. In contrast, the fraction of rRNAs, tRNAs and house-keeping RNAs decreased from 35.2 to 14.6% (MEP) and 47.7 to 23.8% (ESP), respectively. When comparing relative abundances of individual sRNAs (reflected by the number of reads in Hfq-3xFLAG samples), we observed a cell density-dependent shift in the profiles of posttranscriptional regulators in different growth phases. For example, ChiX, GcvB, and Spot42 accounted for more than two-thirds of all reads in rapidly dividing cells, whereas CyaR, ArcZ, and RprA accumulated in cells upon shifting to stationary growth (Fig. 1*D*).

With our approach, we detected 69 conserved enterobacterial sRNAs and annotated 53 candidate sRNAs, 83 of which interacted with Hfq (≥threefold enrichment when compared to the control, see *SI Appendix*, Table S1 and Dataset S2). Regarding a consistent nomenclature, all sRNA candidates without a functional assignment were termed KpnR_NNN.

We classified the sRNAs by their genomic location, i.e., whether they were transcribed from their own promoters in intergenic regions (IGR) or encoded within mRNA 5′UTRs, CDS or 3′UTRs (*SI Appendix*, Fig. S3*A* and Table S1). We identified a high fraction of 75 IGR-encoded sRNAs which are transcribed from their own promoters and in general harbor a Rho-independent terminator, a structural element characterized by a 3′ terminal GC-rich hairpin followed by a single-stranded uridine-stretch. The second most prominent class were 3′UTR-derived sRNAs (29 candidates) released from the rest of the protein-coding message by endonucleolytic cleavage. Hfq-associated mRNA 3′ ends may act as bona fide regulatory RNAs, and well-studied examples include the enterobacterial sRNAs GlnZ, DapZ, and CpxQ (29–33), which are also conserved in *Klebsiella* and enriched in our RIP-seq experiment (Fig. 1*D*, *SI Appendix*, Table S1, and Dataset S2). In addition, we detected 11 5′UTR-derived candidates that may originate from premature transcription termination and associate with Hfq independent of the rest of the message, as well as ten asRNAs.

Northern blot analysis to assess relative expression patterns for selected, uncharacterized sRNA candidates revealed a decrease in sRNA abundance for KpnR_040, KpnR_069, KpnR_127, and KpnR_128 in the absence of Hfq, whereas KpnR_006, KpnR_105, KpnR_117, and KpnR_124 levels were not affected (*SI Appendix*, Fig. S3*B*).

### Hfq RIL-seq Analysis.

To examine the regulatory roles of conserved and candidate sRNAs in *K. pneumoniae*, we performed RIL-seq using Hfq as a bait. Our RIP-seq analysis had revealed highest diversity of sRNAs in ESP (OD_600_ of 2.0; Fig. 1*D*, *SI Appendix*, Table S1, and Dataset S2), and we thus chose this growth phase to globally map Hfq-dependent RNA–RNA interactions. Following the original protocol (34) with minor modifications, we used UV light to cross-link RNA and proteins prior to co-immunoprecipitation of Hfq-3xFLAG. A replicate experiment with the WT strain lacking the 3xFLAG epitope served as control. Upon trimming of the RNA, we fused proximal transcript ends by ligation to obtain RNA chimeras and examined the purified RNA by paired-end sequencing. We recovered single fragments for which both of the two sequences mapped to the same position on the genome, as well as chimeric fragments representing potential RNA–RNA interactions which were excerpted during the sequencing analysis when the inserts of two corresponding reads mapped to different genomic locations.

**Fig. 2.**
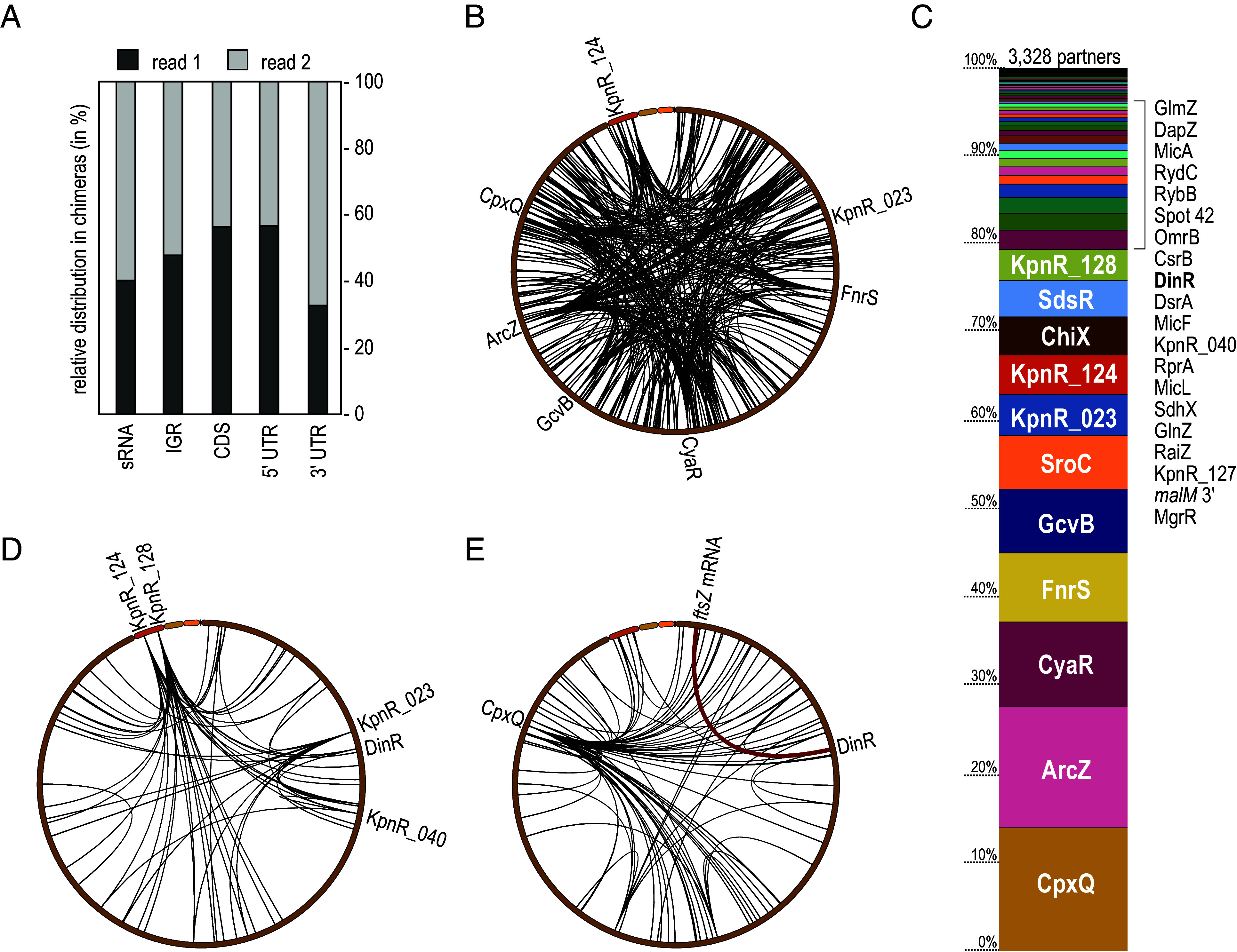
RIL-seq reveals the *K. pneumoniae* Hfq-dependent RNA interactome. (*A*) Relative distribution of RNA classes and their organization in chimeric reads. (*B*) Circos plot of all RIL-seq interactions recovered in both replicates involving *K. pneumoniae* sRNAs. Interaction hubs of selected sRNAs are indicated. (*C*) Relative distribution of numbers of interaction partners in Hfq RIL-seq for all sRNAs. (*D*) Circos plot of all RIL-seq interactions recovered in both replicates involving previously unknown candidate sRNAs. Selected sRNAs with multiple partners are indicated. (*E*) Circos plot of all RIL-seq interactions recovered in both replicates involving sRNA derived from mRNA 3′ ends. CpxQ as an interaction hub is marked. The interaction between DinR and *ftsZ* mRNA is highlighted in red.

Compared to the control, the Hfq-3xFLAG immunoprecipitate revealed a significant enrichment of single and chimeric fragments (*SI Appendix*, Fig. S4*A*). Taken together, we identified 7,068 Hfq-bound RNA–RNA interactions represented by at least five reads from a total number of ~1.7 million chimeric cDNAs recovered from two biological replicates, and more than half of all chimeric reads mapped to at least one sRNA (*SI Appendix*, Fig. S4*B*). As with RIL-seq analyses in other bacterial species (35–37), we also observed numerous sRNA–sRNA chimeras, suggesting that the approach is suitable to recover noncanonical, Hfq-mediated interactions. When analyzing chimera organization, we observed a higher abundance of sRNAs and 3′UTR-derived transcripts in read 2 than in read 1 (Fig. 2*A*), a tendency that has also been observed in other studies (35–39). Binding of the U-rich sequences at the 3′ end of Rho-independent terminators (as found in most sRNAs and a subset of mRNAs) to the proximal surface of Hfq constrains ligation efficiency and results in an enrichment of sRNA sequences linked to their interacting RNAs via the 5′ terminus.

We detected Hfq-mediated interactions with trans-encoded messages for 74 sRNAs, 32 of which have been annotated in this study (Dataset S3). Inspection of all chimeras containing sRNAs represented in both replicates (n = 526) revealed distribution of the mapped sequences throughout the genome (Fig. 2*B*). A few interacting hubs representing sRNAs with an extensive target spectrum dominate the plot, including well-studied examples like ArcZ, CyaR and GcvB (40–44) but also the previously unknown sRNA candidates KpnR_124, KpnR_023, and KpnR_128 (Fig. 2 *B*–*D*). An abundant class of RNA regulators overlap with the 3′ regions of mRNAs, either being released from the mature transcript through endonucleolytic cleavage or being transcribed from their own promoters. 97 Hfq-dependent chimeras involved 3′ end-derived sRNAs of which the most abundant consisted of the *skp* mRNA and CpxQ sRNA (Fig. 2*E* and Dataset S4), a regulatory interaction known to contribute to inner membrane homeostasis in response to stress (33). Among the top-ranked interactions, we furthermore noticed an interaction between the previously unknown sRNA candidate KpnR_029 (derived from the 3′ end of *dinI* mRNA; DinR) and the 5′UTR of the *ftsZ* transcript.

### Processing of the *dinI* mRNA Releases the Hfq-Dependent sRNA DinR.

**Fig. 3.**
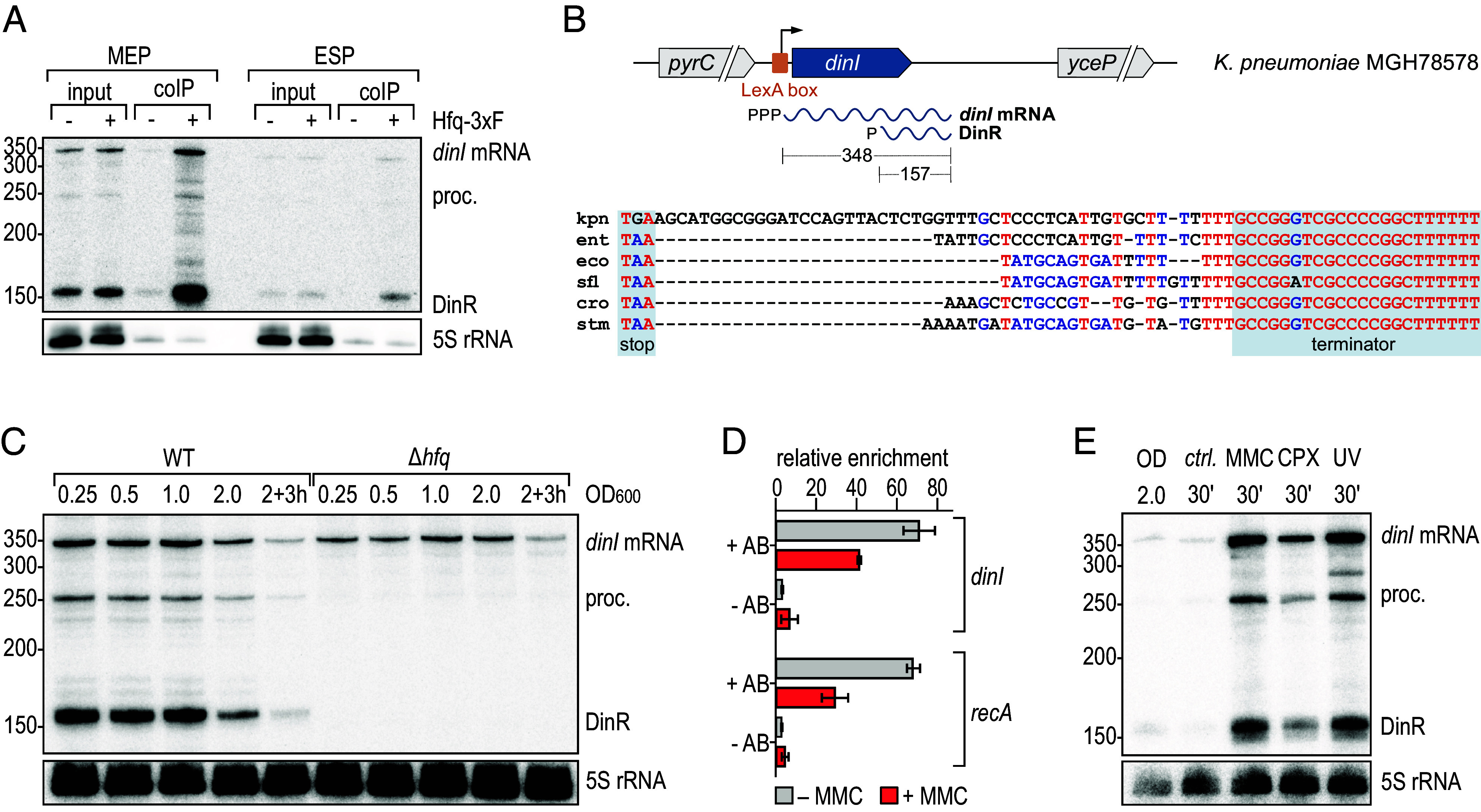
DinR is an Hfq-associated sRNA produced in response to DNA damage. (*A*) RNA samples recovered from the Hfq RIP-seq analysis (Fig. 1) were analyzed on Northern blots and probed with a DinR-specific riboprobe to determine expression and enrichment of *dinI* mRNA and its processing products including DinR. 5S rRNA served as loading control. (*B*) *Upper*: Organization of the *dinI* locus on the main chromosome of *K. pneumoniae* MGH 78578. A LexA-box overlapping the *dinI* TSS is indicated by an orange box. The *dinI* primary transcript (348 nt) is processed, releasing DinR (157 nt). *Lower*: Nonredundant alignment of the *dinI* 3′UTR in diverse enterobacteria (kpn: *Klebsiella pneumoniae* MGH 78578; ent: *Enterobacter sp.* 638; eco: *E. coli* MG1655; sfl: *Shigella flexneri* 301; cro: *Citrobacter rodentium* ICC168; stm: *Salmonella* Typhimurium LT2); the *dinI* stop codon and the Rho-independent transcriptional termination site are shaded in gray. Nucleotides are colored regarding their degree of conservation (red: high conservation; blue: partial conservation; black: little or no conservation). (*C*) Expression of *dinI* mRNA and DinR sRNA in WT and Δ*hfq K. pneumoniae*. RNA samples were collected at different time points over growth (OD_600_ from 0.25 to 2.0, and 3 h after cells had reached OD_600_ of 2.0) and analyzed by Northern blotting. 5S rRNA served as loading control. (*D*) In vivo binding of LexA to the *K. pneumoniae dinI* promoter. Association of LexA before and after addition of MMC was determined by ChIP (+AB: anti-LexA antibody; −AB: no antibody control) followed by quantitative PCR. Relative enrichment of DNA fragments was calculated as a ratio between the tested promoter region and a control region located within the *sgrR* CDS. (*E*) Expression of DinR in response to DNA damage. WT cells were cultivated in LB to OD_600_ of 2.0. RNA samples were collected prior to and 30 min after induction of DNA damage with MMC, CPX or UV, or from an untreated control (*ctrl.*). Expression of *dinI* mRNA and DinR was assessed by Northern blot analysis; 5S rRNA served as loading control. See *SI Appendix*, Fig. S7 for a relative comparison of *dinI* mRNA and DinR expression levels.

The prominent class of sRNAs derived from mRNA 3′ ends frequently function in the same pathways as the protein-coding transcript they are released from (45), and we were curious to investigate the biological role of candidate sRNA KpnR_029. The 83 aa protein DinI encoded in *dinI* mRNA impacts RecA filamentation to modulate induction and shut-off of the SOS response (46). Comparison of *dinI* sequences indicated high conservation of the gene at the nucleotide and amino acid level (*SI Appendix*, Fig. S5 *A* and *B*), but also uncovered a *Klebsiella*-specific sequence stretch upstream of the Rho-independent terminator (Fig. 3*B* and *SI Appendix*, Fig. S5*A*). Northern blot analysis of total RNA and RNA co-immunoprecipitated Hfq-3xFLAG of *dinI* mRNA revealed a ~350 nt transcript representing the full-length mRNA as well as two shorter fragments (Fig. 3 *A* and *B*) for which we mapped the respective 5′ ends employing our dRNA-seq and Hfq RIP-seq datasets, as well as primer extension analysis (*SI Appendix*, Fig. S6). All three transcript isoforms were expressed during growth of *K. pneumoniae* in LB medium, with a decrease toward the stationary phase (Fig. 3*C*). In the absence of Hfq, only the full-length transcript was detectable. As prompted by the presence of a LexA box overlapping the TSS (Fig. 3*B* and *SI Appendix*, Fig. S5*A*), we anticipated that *dinI* expression was triggered by DNA damage. Chromatin-immunoprecipitation (ChIP) using a LexA-specific antibody confirmed reduced occupancy of the *dinI* promoter by LexA upon exposure of *K. pneumoniae* to mitomycin C (MMC) (Fig. 3*D*). In accordance with this finding, treatment with MMC, ciprofloxacin (CPX), or irradiation with ultraviolet light (UV), resulted in an induction of full-length *dinI* mRNA as well as an accumulation of the two shorter fragments (Fig. 3*E*). Reflecting its origin in the 3′ end of *dinI*, we renamed the abundant, 157 nt Hfq-bound sRNA candidate DinR. As we were unable to annotate a potential promoter upstream of either of the two shorter transcripts we suspected that the *dinI* mRNA was being processed. To test this hypothesis, we cloned *dinI* either including ~50 bp upstream the mapped TSS, or a promoterless version on a multicopy plasmid. We transformed the constructs into *dinI* mutant *K. pneumoniae* and compared *dinI* mRNA and DinR expression upon exposure of the cells to MMC to a WT strain carrying an empty control vector. Northern blot analysis confirmed that the *dinI* promoter was required for the expression of full-length *dinI* mRNA as well as DinR, suggesting that the shorter transcripts are processing products (*SI Appendix*, Fig. S8*A*). The conserved endoribonuclease E (RNase E) is central to RNA processing and decay in enterobacteria and involved in the release of many Hfq-dependent sRNAs from mRNA 3′ ends (45). We tested the requirement for RNase E for the production of DinR employing a *Salmonella* strain carrying a temperature-sensitive allele of the essential *rne* gene (*rne*TS; *rne-3071*) and its isogenic control strain expressing wt *rne*. Both strains exhibit full RNase E activity and DinR production when cultivated at 30 °C. Upon shifting the *rne*TS-strain to the nonpermissive temperature for RNase E activity of 44 °C the DinR signal disappeared and solely full-length *dinI* mRNA was detectable (*SI Appendix*, Fig. S8*B*). Based on these results, we conclude that *dinI* is processed by RNase E at position 169 within the CDS to release the DinR sRNA.

### DinR Is an Hfq-Dependent Trans-Acting sRNA.

**Fig. 4.**
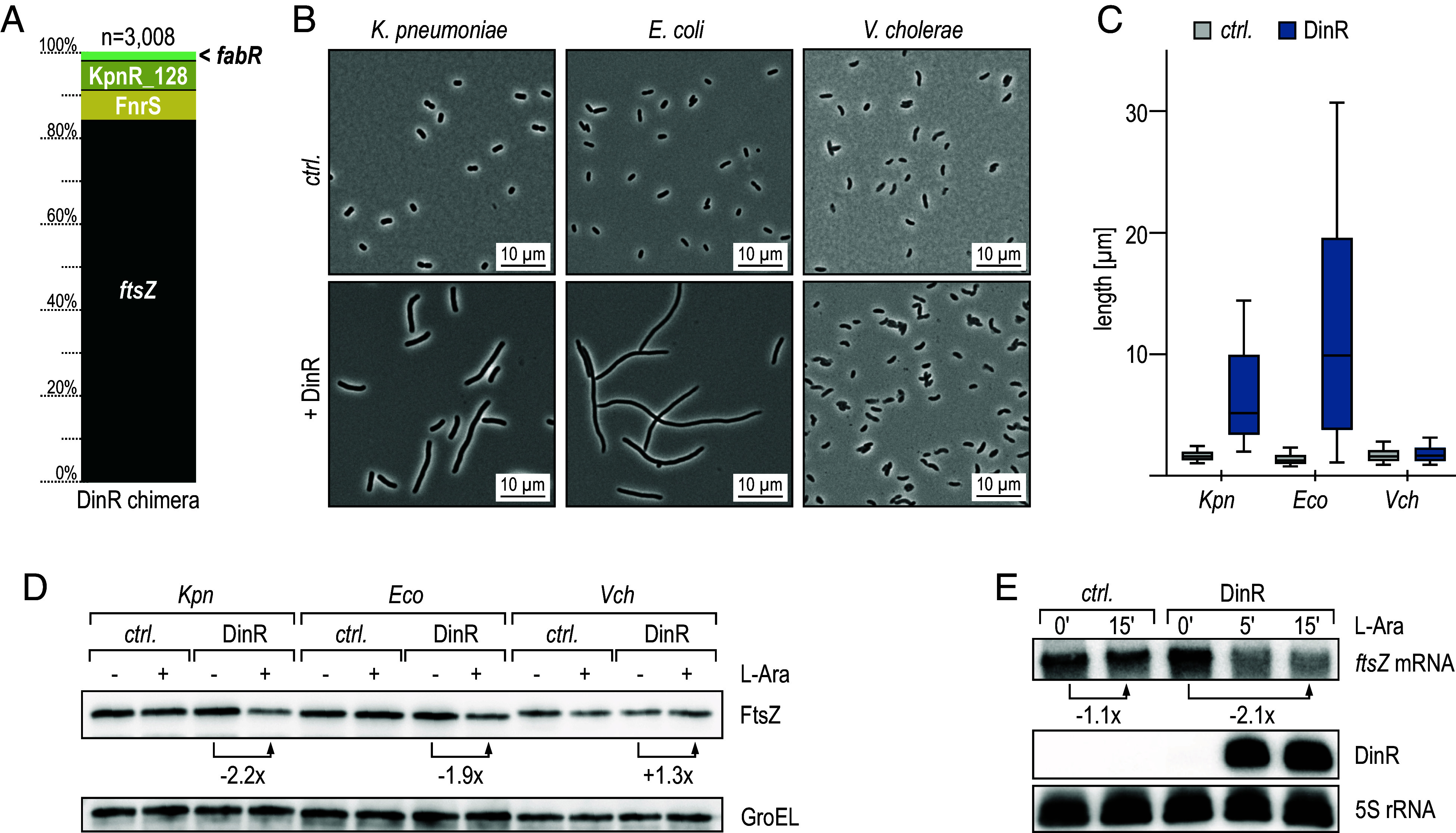
RIL-seq determines *ftsZ* mRNA as the main target of DinR. (*A*) Relative distribution of RIL-seq chimeras of DinR represented in both replicates. (*B*) *K. pneumoniae*, *E. coli,* and *V. cholerae* carrying either an empty control vector (pBAD_KP_-*ctrl.*, pBAD_EC_-*ctrl.*, or pBAD_VC_-*ctrl.*, respectively) or pBAD variants for the expression of DinR (pBAD_KP_-DinR, pBAD_EC_-DinR, or pBAD_VC_-DinR, respectively) were diluted from overnight cultures into fresh medium, and sRNA expression was induced by the addition of arabinose. Cell morphology was assessed by phase contrast microscopy after 5 h. (*C*) Analysis of cell lengths in samples described in (*B*). The center line indicates the median, boxes represent the 25th and 75th percentiles, and lower and upper whiskers represent the 10th and 90th percentiles, respectively. (*D*) FtsZ protein levels were determined by western blot analysis using a FtsZ-specific antiserum in total protein samples collected from strains described in (*B*), which were diluted from overnight cultures into fresh medium, and cultivated for 5 h in the absence (−) or presence of arabinose (+). GroEL served as loading control. (*E*) *K. pneumoniae* carrying either pBAD_KP_-*ctrl.* or pBAD_KP_-DinR were grown to OD_600_ of 2.0 when expression from the *araBAD* promoter was induced by the addition of arabinose. Expression of DinR and *ftsZ* mRNA was determined by Northern blot analysis of RNA samples collected at indicated time points. 5S rRNA served as loading control.

Our RIL-seq analysis recovered several interactions with DinR (Figs. 2*E* and 4*A*); however, the by far most abundant pair was formed with the 5′ end of the *ftsZ* mRNA, encoding the essential bacterial tubulin homologue. Upon localization to the division plane, FtsZ polymerizes to form the Z-ring, a scaffold structure to which additional cell division proteins are recruited and which guides septal peptidoglycan synthesis to mediate constriction (47). To prevent cell division prior to DNA repair, FtsZ polymerization is inhibited by the SOS response in *E. coli* through the LexA-regulated SulA, resulting in the characteristic cell filamentation (48).

The cellular FtsZ protein levels need to be accurately balanced as a reduction by ≥30% causes cell elongation and inhibition of division while a ≥twofold increase results in mini-cell formation (49). To test the effect of DinR on *ftsZ* expression, we performed a microscopy-based screen to monitor cell morphology. Overexpression of the *K. pneumoniae* sRNA DinR from an arabinose-inducible promoter resulted in highly elongated cells and reduced FtsZ protein levels in both *Klebsiella* and *E. coli* by ~50% but did not affect *Vibrio cholerae* FtsZ (Fig. 4 *B*–*D* and *SI Appendix*, Fig. S9*A*), pointing at potent inhibition of FtsZ production by the sRNA in the enterobacterial species. Comparison of the genomic sequences upstream of the *ftsZ* start codon between the three species revealed significant differences in *V. cholerae* (*SI Appendix*, Fig. S9*B*), hinting at an interaction between DinR and the mRNA within its 5′UTR. To verify that DinR repression of *ftsZ* occurred at the RNA level, we pulse-expressed the sRNA and quantified *ftsZ* mRNA levels by Northern blot analysis. Compared to the control, induction of DinR resulted in twofold down-regulation of the *ftsZ* transcript, suggesting that the reduction in FtsZ protein in the presence of DinR was the result of mRNA repression (Fig. 4*E*).

**Fig. 5.**
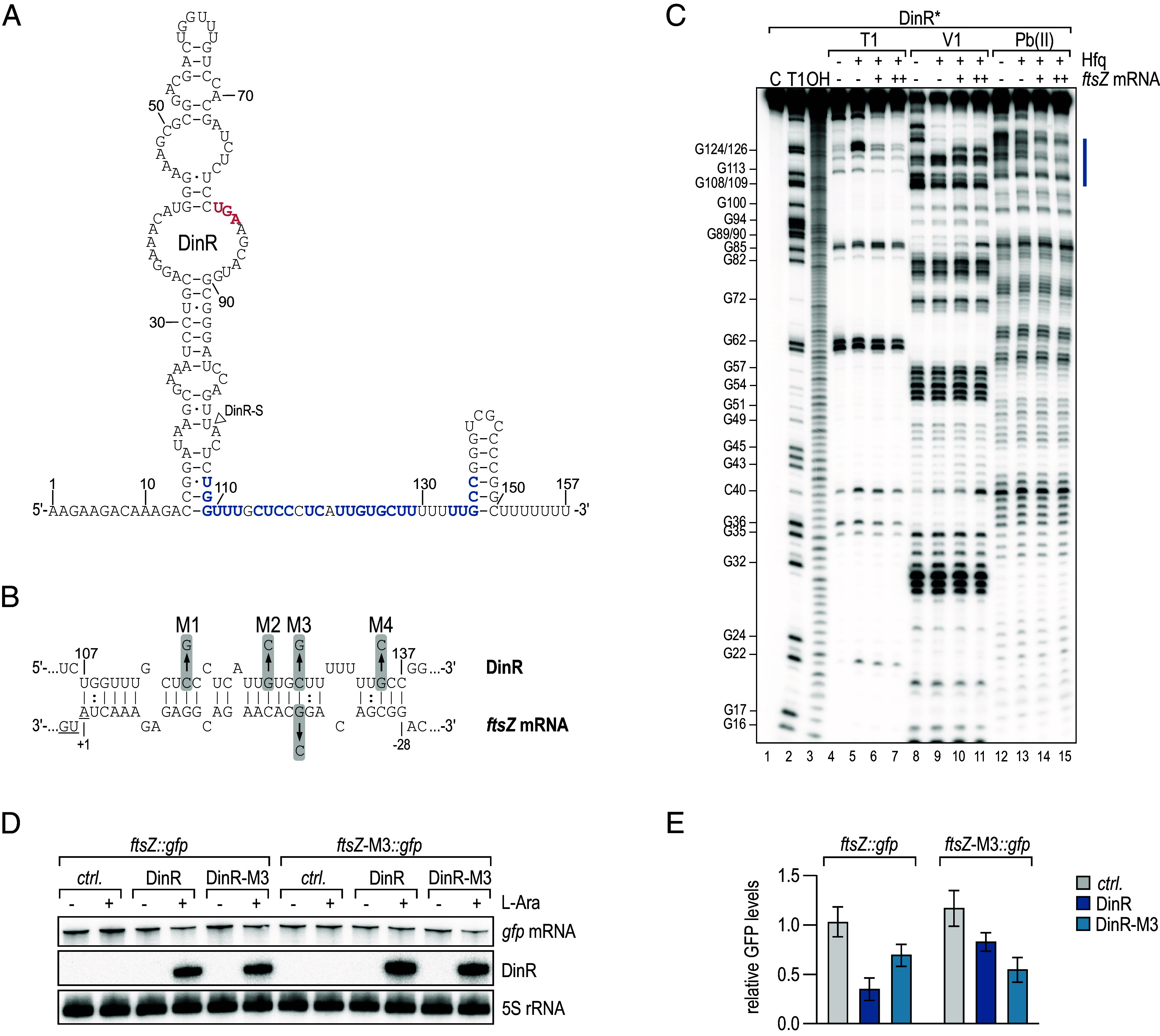
DinR represses *ftsZ* mRNA through a direct RNA–RNA interaction. (*A*) Secondary structure of DinR as determined by bioinformatics predictions and chemical probing shown in (*C*). The *dinI* stop codon is marked in red; the nucleotides predicted to interact with *ftsZ* mRNA are highlighted in blue. The 5′ end of DinR-S is indicated by a triangle. (*B*) Predicted base-pairing interaction forming between DinR and *ftsZ* mRNA. For DinR, the positions are numbered relative to the sRNA start site. For *ftsZ* mRNA, the positions are numbered relative to the start codon (underlined). Positions of single-nucleotide exchanges generating mutants M1, M2, M4, and the compensatory mutant M3 are indicated. (*C*) In vitro structure probing of 5′-end-labeled DinR (0.4 pmol) sRNA with RNase T1 (lanes 4 to 7), RNase V1 (lanes 8 to 11), and lead(II) acetate (lanes 12 to 15) in the absence (−) or presence (+) of 5x Hfq protein and 10x (+) or 25x (++) *ftsZ* mRNA. RNase T1 and alkaline ladders of DinR were used to map the positions of individual nucleotides. The putative *ftsZ* mRNA binding site is marked in blue. (*D*) *E. coli* carrying either an empty control vector pBAD_EC_-*ctrl.*, pBAD_EC_-DinR or pBAD_EC_-DinR-M3 in combination with the posttranscriptional reporters *ftsZ::gfp* or *ftsZ*-M3*::gfp*, respectively, were cultivated to OD_600_ of 0.5, and then, expression from the *araBAD* promoter was induced. RNA samples collected prior to and 5 min after addition of arabinose were analyzed by Northern blotting to determine the expression of DinR and *ftsZ::gfp* mRNA and their respective variants; 5S rRNA served as loading control. (*E*) Quantification of *ftsZ::gfp* or *ftsZ*-M3*::gfp* mRNA levels 5 min after addition of arabinose determined as described in (*D*). mRNA levels were determined relative to the expression levels prior to addition of the inducer; error bars represent the SD calculated from three independent biological replicates.

Using the RNAhybrid algorithm (50), we identified an extensive interaction between the *ftsZ* 5′UTR in proximity to the start codon and a sequence stretch upstream of the DinR terminator (Fig. 5 *A* and *B*). Probing of in vitro synthesized, 5′-end-labeled DinR sRNA with RNases T1 and V1, or lead(II), revealed specific protection of the predicted site in the presence of *ftsZ* mRNA and Hfq (Fig. 5*C*). In a reciprocal experiment, we also confirmed the expected base-pairing site of DinR on the *ftsZ* 5′UTR (*SI Appendix*, Fig. S10). We introduced a series of point mutations in DinR at different sites of the interaction (M1 to M4; Fig. 5*B*) and queried the potential of the individual sRNA variants to interact with *ftsZ* mRNA in vivo and in vitro. Based on the protection pattern, base-pairing efficiency of mutants M2 and M3 was strongly reduced when compared to WT DinR, whereas mutants M1 and M4 were still proficient to interact with the *ftsZ* mRNA (*SI Appendix*, Fig. S10). When scoring filamentation upon overexpression of either of the DinR variants in *Klebsiella*, we observed partial reduction of the cell elongation phenotype in the presence of M1, M2, and M3, suggesting that these residues were involved in base-pairing with *ftsZ* (*SI Appendix*, Fig. S11 *A* and *B*). Moreover, overexpression of M4 did not result in filamented cells, contradicting the result obtained with purified components in vitro. Determination of sRNA abundance by Northern blot analysis, however, revealed poor expression of DinR-M4 compared to the other variants (*SI Appendix*, Fig. S11*C*), explaining its inability to down-regulate *ftsZ* mRNA in vivo. We constructed a posttranscriptional reporter in which the 5′UTR plus the first 20 codons of *ftsZ* fused to *gfp* are expressed from a constitutive promoter. As the filamentation phenotype impeded a reporter assay involving long-term overexpression of DinR, we instead pulse-expressed the sRNA for 5 min and quantified *ftsZ::gfp* mRNA levels by Northern blot analysis. Introduction of a SNP in either of the two interacting RNAs (DinR-M3 or *ftsZ*-M3*::gfp*) abrogated the inhibitory effect of the sRNA, and restoration of the RNA duplex by complementary mutations of both sRNA and mRNA rescued *ftsZ* regulation (Fig. 5 *D* and *E*). Collectively, our data suggest that DinR acts as an Hfq-dependent posttranscriptional repressor of the *ftsZ* mRNA.

### DinR Contributes to Cell Cycle Arrest of *Klebsiella* in Response to DNA Damage.

**Fig. 6.**
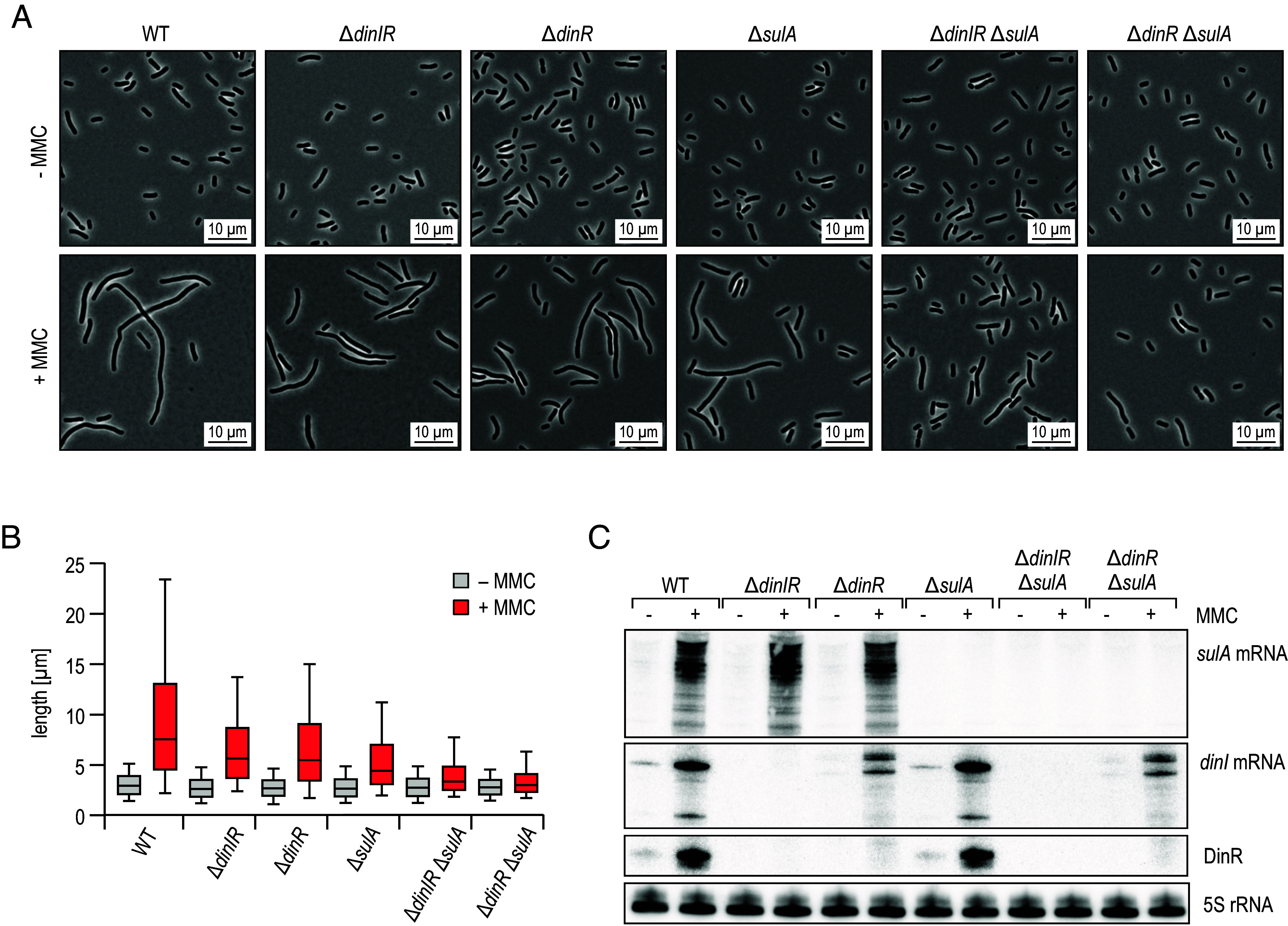
DinR and SulA contribute to cell filamentation in response to DNA damage. (*A*) *K. pneumoniae* WT, Δ*dinIR*, Δ*dinR,* Δ*sulA,* Δ*dinIR* Δ*sulA*, and Δ*dinR* Δ*sulA* cells were diluted from overnight cultures into fresh medium, and grown for 30 min. Cultivation was continued for 5 h in the presence (+) or absence (−) of MMC to induce DNA damage. Cell morphology was assessed by phase contrast microscopy. Details on mutant design at the *dinIR* locus are provided in *SI Appendix*, Fig. S12*A*. (*B*) Analysis of cell lengths in samples described in (*A*). The center line indicates the median, boxes represent the 25th and 75th percentiles, and lower and upper whiskers represent the 10th and 90th percentiles, respectively. (*C*) RNA samples were collected from cells cultivated as described in (*A*) for 30 min in the presence (+) or absence (−) of MMC.

**Fig. 7.**
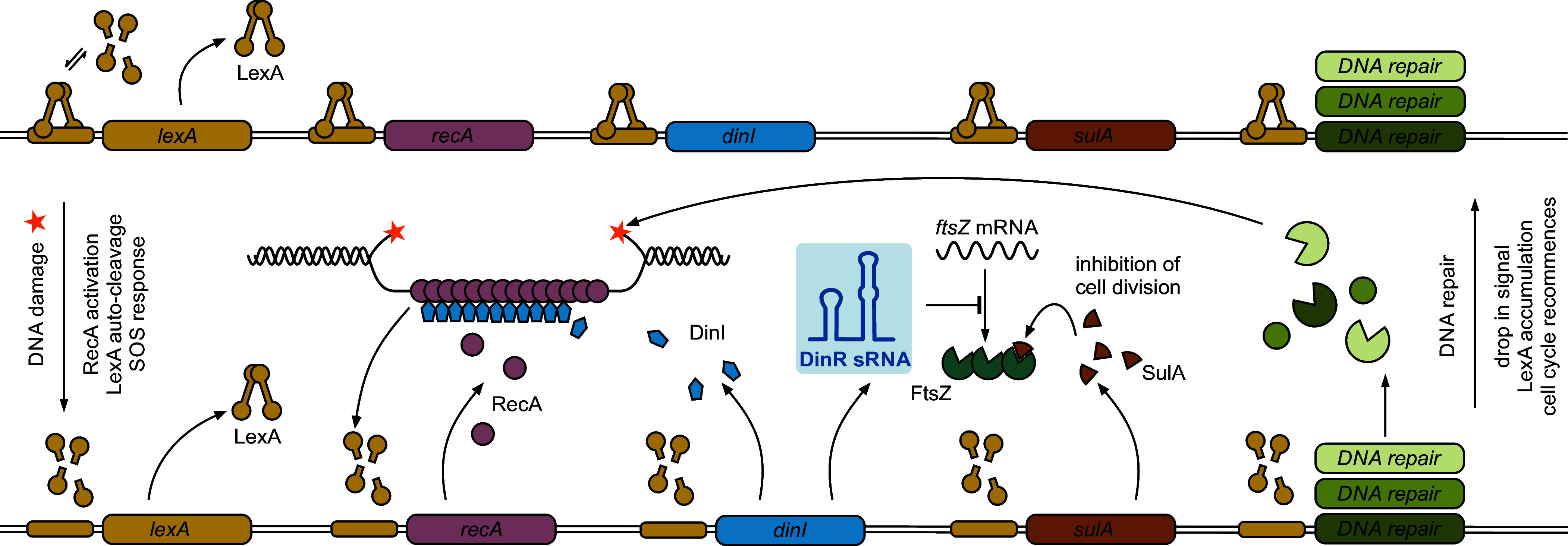
DinR sRNA functions in the SOS response to DNA damage. In the absence of DNA damage (*Upper* part), LexA repressor dimers are bound to operator sequences (SOS boxes) within the promoters of SOS genes. Under SOS-inducing conditions (*Lower* part), RecA assembles on ssDNA to form a nucleoprotein filament which catalyzes LexA autoproteolysis, resulting in SOS gene derepression. Repair pathways of different fidelity are induced to ensure removal of DNA lesions. SulA protein constrains FtsZ activity and Z-ring formation to allow the bacterium to repair its DNA prior to cell division. In this study, we identify the Hfq-dependent sRNA DinR as an additional inhibitor of cytokinesis in *K. pneumoniae*, repressing *ftsZ* mRNA at the posttranscriptional level through a direct base-pairing interaction. DinR is processed from the 3′ end of *dinI* mRNA, encoding for a small protein modulating RecA filament stability. With *lexA* being a SOS gene itself, a negative-feedback loop ensures re-establishment of LexA-mediated repression once the stress signal is ceased.

DinR levels strongly increase in response to DNA damage in a LexA-dependent manner (Fig. 3 *D* and *E*). As SulA has been documented to inhibit FtsZ function at the protein level under this condition (13, 14), we hypothesized that DinR could add to this regulation through posttranscriptional repression of the *ftsZ* mRNA. *Klebsiella* mutant strains lacking either *sulA*, *dinIR*, *dinR* alone or in combination were not affected in cell morphology in the absence of DNA damage (Fig. 6 *A*, *Top* and Fig. 6*B*). We next treated the cells during exponential growth with MMC to induce DNA damage, and Northern blot analysis confirmed activation of *sulA* and *dinIR* under this condition (Fig. 6*C*). Cell morphology was analyzed by phase contrast microscopy after 5 h, and the degree of filamentation in each mutant was determined by measuring cell lengths. As observed in other bacterial species (51), the SOS response of *K. pneumoniae* is heterogeneous and we detected a distribution of cell sizes within the population. As expected, the mean cell length in *sulA* mutants was decreased when compared to the WT, however, did not recover the short cell morphology of cells observed in the absence of DNA damage (Fig. 6 *A* and *B*). Deletion of either *dinIR* or *dinR* alone also resulted in partial reduction of the filamentous phenotype. To better separate the individual functions of DinI protein and the sRNA DinR, we complemented the *dinIR* double mutant with constructs expressing the WT allele or a derivative in which the *dinI* start codon had been inactivated (*SI Appendix*, Fig. S12*B*). Both variants fostered a similar increase in cell length, suggesting that the protein coding function of *dinIR* was not essential for cell elongation (*SI Appendix*, Fig. S12*C*). Cells carrying a third construct in which the base-pairing site of DinR was missing phenotypically resembled the *dinIR* mutant strain. We measured the most severe reduction of cell elongation when cells were unable to express neither *dinR* nor *sulA*, suggesting that DinR and SulA may work together to efficiently inhibit cell cycle progression during the SOS response (Figs. 6 *A* and *B* and 7).

## Discussion

Bacterial pathogenesis is critically dependent on the microorganism’s ability to respond to environmental changes and host-specific cues. Complex networks of stress adaptation warrant accurate and timely rewiring of gene expression, and sRNAs have herein been recognized as key posttranscriptional regulators. Work in enteric pathogens, including *Salmonella* and uropathogenic *E. coli*, has revealed a critical role for Hfq in the establishment of an infection and mutation of the *hfq* gene has been associated with pleiotropic phenotypes (52, 53). Although it had previously been shown that *hfq* mutants of *K. pneumoniae* are impaired in virulence and stress resilience (23), the RNA ligands of Hfq and the network of RNA-mediated regulation in this organism were unidentified. In this study, we have combined three high-throughput analyses to establish a global atlas of sRNA activity in the multidrug-resistant *K. pneumoniae* isolate MGH 78578. A high-resolution transcriptome map obtained through dRNA-seq in combination with Hfq co-immunoprecipitation of RNA enabled us to chart conserved as well as numerous candidate sRNAs. On this basis, we have applied RIL-seq to unearth an intricate network of sRNAs and their RNA interaction partners and identified DinR, a 3′UTR-derived, Hfq-dependent sRNA as an inducer of cell filamentation in the SOS response elicited by DNA damage.

### sRNAs Functioning in the SOS Pathway and the Response to DNA Damage.

The SOS response is an integral part of the defense against DNA-damaging agents in bacteria (8). Activation of the SOS response has been reported to result in the expression of several noncoding regulators, which are part of toxin-antitoxin systems and thus are bona fide *cis*-acting regulators (54). In contrast, DinR is an Hfq-dependent (i.e., *trans*-acting) sRNA that is activated by LexA, the master regulator of the SOS response. Further inspection of the 3,748 TSS identified in our dRNA-seq dataset revealed potential LexA binding sites upstream of 71 transcription units (*SI Appendix*, Table S2), 25 of which have also been reported for *E. coli* (10, 55). Six genes linked to an SOS box were enriched greater than threefold in at least one condition in the Hfq coIP samples; however, none of them was associated with an sRNA gene except for *dinIR* (*SI Appendix*, Table S2). While *dinI* is highly conserved in the enterobacteria (*SI Appendix*, Fig. S5), it remains to be determined whether processing also releases a stable fragment from the mRNA 3′ end in other species which could act as a regulatory RNA alike *Klebsiella* DinR. Of note, the stretch of DinR predicted to base-pair with *ftsZ* mRNA spans the highly variable region of the *dinI* 3′UTR specific to *Klebsiella* (Fig. 3*B* and *SI Appendix*, Fig. S5*A*).

Despite the lack of other sRNAs directly regulated by LexA, sRNAs are well known to respond to DNA-damaging conditions and antibiotic treatment. For example, the highly conserved GcvB sRNA affects homology-directed mutagenic DNA break repair (MBR) in *E. coli* by modulating the competition between the two alternative sigma factors RpoE and RpoS (56). RpoS is required for MBR activation (7) and subject to complex transcriptional, posttranscriptional, and posttranslational control. Three sRNAs, i.e., DsrA, RprA, and ArcZ, have been described to increase *rpoS* translation in response to various stress conditions, including the fluoroquinolone antibiotics which activate DsrA and ArcZ expression (57, 58). ArcZ, as well as the Hfq-binding sRNA SdsR, also inhibit the expression of the *mutS* mRNA, encoding an essential component of the mismatch repair system (59, 60). Transcription of *sdsR* is induced by RpoS (61) and mutation of either *rpoS* or *sdsR* reduced the frequency of mutations conferring antibiotic resistance in *E. coli* (60). Taken together, there is accumulating evidence that base-pairing sRNAs play important roles in the response, repair, and defense mechanisms associated with DNA damage in bacteria and it is likely that RNA interactome studies involving global approaches such as RIL-seq and CLASH will further extend this view over the next few years. Of note, noncoding RNAs, called damage-induced small RNAs, are also involved in DNA repair processes in eukaryotic organisms (62), implying that gene regulation at the posttranscriptional level can help maintain genome integrity across the domains of life.

### DinR Inhibits Cytokinesis through Posttranscriptional Repression of *ftsZ* mRNA.

Activation of the SOS pathway triggers a graded, multilayered stress response that involves over 50 genes (63). Following DNA damage and derepression of the LexA-controlled regulon, two main pathways of the SOS response are activated: DNA repair and cell division inhibition. Filamentation of the cell as a consequence of the latter is a hallmark of the bacterial SOS response; however, the underlying mechanisms show significant variation in individual species (48). For instance, in *Caulobacter crescentus*, the small inner membrane protein SidA interferes with the activity of the late cell division proteins FtsN, FtsI, and FtsW, thereby inhibiting the final constriction of the Z-ring (64). In contrast, in the gram-positive bacteria *Bacillus subtilis* and *Staphylococcus aureus*, the SOS-induced YneA and SosA proteins, respectively, promote cell elongation by acting on currently unknown components of the divisome (65, 66).

In *E. coli* and related species, SOS-associated cell filamentation has been documented to rely on the LexA-controlled cytosolic protein SulA, which impedes FtsZ polymerization and thus promotes cell elongation (14). Our work uncovered an additional layer of SOS-dependent FtsZ regulation by the Hfq-dependent sRNA DinR. In contrast to SulA, DinR acts at the posttranscriptional level and represses translation of the *ftsZ* mRNA by directly base-pairing in the vicinity of the ribosome binding site (Fig. 5). Thus, both SulA and DinR inhibit cell division through FtsZ; however, the underlying regulatory mechanisms differ. Interestingly, the SulA protein can be rapidly degraded by the Lon and ClpYQ protease, allowing immediate resuscitation of cell division when the SOS pathway is inactivated (67–69). We currently do not know whether similar mechanisms exist to counteract DinR activity upon stress activation; however, our RIL-seq data suggest that DinR also base pairs with other transcripts, which could help to release the inhibitory effect of DinR on *ftsZ*. The dialogue between the two regulators SulA and DinR acting at different functional levels and the temporal control of their activities within the SOS response is a promising direction for future analyses.

### RNA-Mediated Regulation of FtsZ Expression.

Transcriptional control of *ftsZ* in *E. coli* has been associated with several independent promoters and is subject to regulation by the secondary messenger guanosine 5′-diphosphate 3′-diphosphate (ppGpp) (70, 71). Posttranscriptional regulation of *ftsZ* is similarly complex and involves several *cis*- and *trans*-acting sRNAs. Specifically, three isoforms produced from consecutive promoters have been reported for the StfZ RNA that is located antisense to the *ftsZ* gene (72, 73). Although StfZ overexpression reduces FtsZ protein levels, physiological conditions resulting in differential expression of the sRNA have not been identified, suggesting that StfZ might function by fine-tuning *ftsZ* mRNA levels, rather than blocking cell division in response to a distinct stress (72).

Another sRNA known to control cell division by base-pairing with the *ftsZ* mRNA is DicF which is processed from a polycistronic message of the cryptic *E. coli* Qin prophage and accumulates under oxygen-limited conditions (74–76). Analogous to DinR, DicF down-regulates *ftsZ* translation by sequestration of the mRNA’s Shine-Dalgarno sequence, and overexpression of the sRNA results in cell filamentation (77). DicF also inhibits the expression of several additional genes involved in metabolic regulation; however, it is not clear how these functions tie together with the regulation of cell division via *ftsZ* (78).

A third sRNA reported to modulate FtsZ levels is OxyS. In contrast to DinR, StfZ, and DicF, OxyS does not act by base-pairing to the *ftsZ* mRNA, but rather interacts with the mRNA encoding the elongation factor NusG (79). Translational inhibition of *nusG* by OxyS results in derepression of the prophage-encoded KilR small protein, which in turn blocks FtsZ polymerization (79, 80). Beyond the known examples, global RNA-RNA interactome studies may in the future reveal an even more complex network of post-transcriptional regulation of FtsZ levels by sRNAs that function under various physiological conditions using a diverse set of regulatory mechanisms.

## Materials and Methods

### Bacterial Strains and Plasmids.

All strains, plasmids, and oligonucleotides used in this study are listed in *SI Appendix*, Tables S3–S5. Details on strain and plasmid construction are provided in *SI Appendix*, *Supporting Methodology*.

### RNA Isolation and Northern Blot Analysis.

Total RNA was prepared using the Hot Phenol method as described previously (81). For Northern blot analysis, 5 to 10 µg of total RNA were separated on 5 to 8 % polyacrylamide gels and electroblotted. Membranes were hybridized with 5′ end-labeled DNA-oligonucleotides at 42 °C or with riboprobes at 68 °C and washed in three subsequent steps with SSC wash buffers (5x/1x/0.5x SSC) supplemented with 0.1 % SDS.

### dRNA-seq and TSS Prediction.

Total RNA was prepared in biological duplicates from *K. pneumoniae* WT cells grown to MEP (OD_600_ of 0.25) or to early stationary phase (ESP; OD_600_ of 2), respectively, and subjected to dRNA-seq analysis (27). Libraries for Illumina sequencing of cDNA were constructed by vertis Biotechnologie AG, Germany. Details on sample preparation and processing of sequencing data are provided in *SI Appendix*, *Supporting Methodology*.

### Hfq RIP-seq.

Duplicates of *K. pneumoniae* WT and *hfq::3xFLAG* strains were cultivated in LB medium to MEP (OD_600_ of 0.25) and ESP (OD_600_ of 2), respectively, and Hfq co-immunoprecipitation was performed as described previously (82). cDNA libraries were prepared from equal RNA amounts using the NEBNext Small RNA Library Prep Set for Illumina (NEB; E7300L) according to the manufacturer’s instructions. cDNA libraries were pooled and sequenced by vertis Biotechnology AG, Germany using an Illumina NextSeq 500 system with 1 × 75 bp read length. Details on the processing of sequencing data are provided in *SI Appendix*, *Supporting Methodology*.

### RIL-seq Experimental Procedures.

Duplicates of *K. pneumoniae* WT and *hfq::3xFLAG* strains were cultivated in LB medium to OD_600_ of 2, and samples were processed following the original RIL-seq protocol (34); a previously published protocol (83) was adapted for the strain-specific depletion of rRNA. Details on the processing of sequencing data are provided in *SI Appendix*, *Supporting Methodology*.

### Microscopy and Image Analysis.

Bacteria were imaged on a Zeiss Axio Imager.Z2 using a 64x oil immersion objective. For quantification, pictures were automatically analyzed using the ImageJ-based visual programming language JIPipe (84). Details on the workflow and its individual components, all results, as well as the utilized software versions are provided in *SI Appendix*, *Supporting Methodology*.

### Protein Sample Analysis.

Whole-protein samples were collected and analyzed by western blotting as described previously (81). 3xFLAG-tagged fusion proteins were detected using a monoclonal anti-FLAG antibody (1:1,000; mouse; Sigma #F1804), and FtsZ levels were probed with a polyclonal antiserum (MJV8; 1:10,000; rabbit; kindly provided by Miguel Vicente, CSIC Madrid). RNAP or GroEL served as loading control and were probed with anti-RNAP (BioLegend; #WP003) or anti-GroEL (Merck; #G6532) antibodies, respectively. Signals were visualized on a Fusion FX imager (Vilber).

### RNA Structure Probing.

RNA structure probing and mapping of Hfq/RNA footprints was conducted as described previously (85) with some alterations. Briefly, 5′ end-labeled RNA was denatured and mixed with *E. coli* Hfq (kindly provided by Kasia Bandyra and Ben Luisi, University of Cambridge) or Hfq dilution buffer in the presence of 1X structure buffer and 1 μg yeast RNA (Invitrogen, #AM7118) and incubated at 37 °C for 15 min. Subsequently, unlabeled sRNA or water was added, and reactions were incubated at 37 °C for another 15 min. After RNase T1 or lead (II) acetate treatment, reactions were stopped by addition of precipitation buffer. RNA was precipitated, washed, and separated by denaturing PAGE.

## Supplementary Material

Appendix 01 (PDF)

Dataset S01 (XLSX)

Dataset S02 (XLSX)

Dataset S03 (XLSX)

Dataset S04 (XLSX)

## Data Availability

Sequencing data have been deposited in the Gene Expression Omnibus at NCBI under accession number GSE244640 (86). The results of the dRNA-seq (including mapped TSSs) and the Hfq RIP-seq experiments are accessible online (https://kp-rnaseq.uni-jena.de/?config=MGH78578/config.json) (87). The RIL-seq data have been implemented into an interactive browser (https://kp-interactome.uni-jena.de/) (88). Details on image analysis are provided at https://asbdata.hki-jena.de/RuhlandEtAl2023_PNAS (89). All data generated or analyzed during this study are included in the manuscript and supporting information.
