# Supplementary material for: The global RNA–RNA interactome of Klebsiella pneumoniae unveils a small RNA regulator of cell division
Source: Proc Natl Acad Sci U S A. 2024 Feb 20;121(9):e2317322121. doi: 10.1073/pnas.2317322121 (PMC10907235; doi:10.1073/pnas.2317322121)
Supplement: Supplementary file 1 — Appendix 01 (PDF) [file pnas.2317322121.sapp.pdf]

PNAS

## Supporting Information for

# The global RNA-RNA interactome of *Klebsiella pneumoniae* unveils a small RNA regulator of cell division

Eric Ruhland, Malte Siemers, Ruman Gerst, Felix Späth, Laura Nicole Vogt, Marc Thilo Figge, Kai Papenfort, Kathrin Sophie Fröhlich

corresponding author: Kathrin Sophie Fröhlich
Email: kathrin.froehlich@uni-jena.de

**This PDF file includes:**

Figures S1 to S12
Supporting Methodology
Tables S1 to S5
SI References

## Supplementary Figure S1

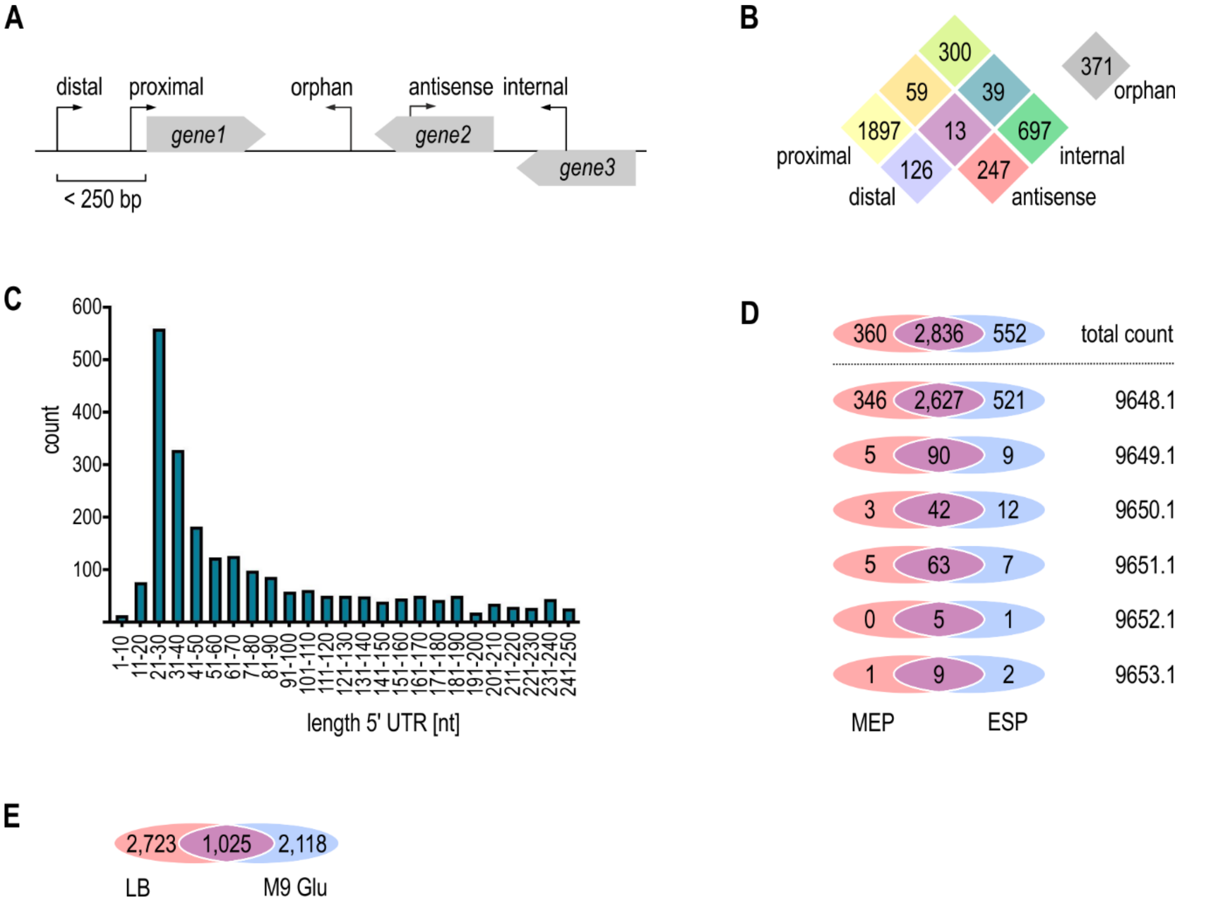


**Annotation and classification of transcriptional start sites (TSSs) in *K. pneumoniae* MGH 78578*.*** (A) TSSs were categorized according to their genomic positions. Proximal and distal TSSs are associated with a gene if located ≤ 250 bp upstream of the start codon on the same strand. Antisense TSSs are located on the opposite strand of an annotated gene, internal TSSs within an annotated gene on the sense strand. A TSS not assigned to any of these categories is classified as orphan. (B) Distribution and overlap of classification for 3,748 TSSs identified in this study. (C) Distribution of the lengths of 5' UTRs based on proximal and distal TSSs. (D) Overlap (purple) among TSSs detected in MEP (red) and ESP (blue) distributed between the core genome (9648.1) and the five plasmids (9649.1; 9650.1; 9651.1; 9652.1; 9653.1) in *K. pneumoniae* MGH 78578*.* (E) Overlap (purple) among TSSs detected in *K. pneumoniae* MGH 78578 in this study for cells grown in LB (red) or in (1) for cells cultivated in minimal M9 medium supplemented with glucose (blue).

## Supplementary Figure S2

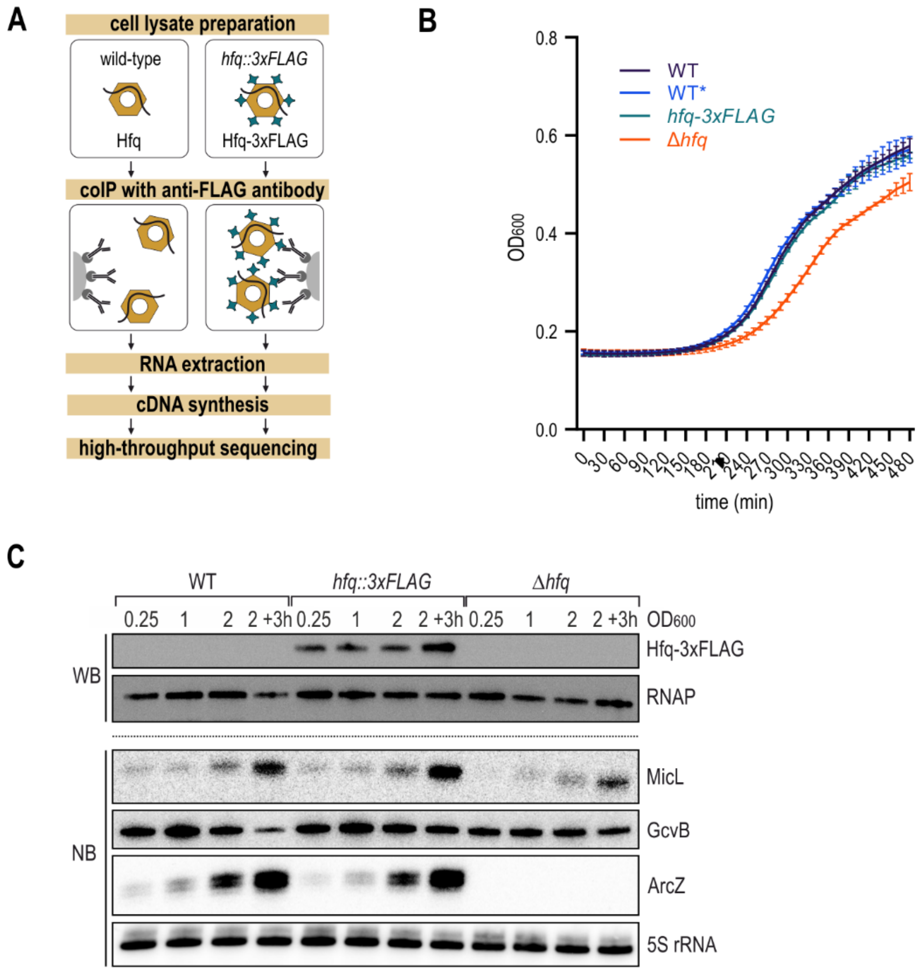


**RNA co-immunoprecipitation and expression profiles of *K. pneumoniae*.** (A) Strategy to identify Hfq-associated RNA. RNA was co-immunoprecipitated from *K. pneumoniae* (wild-type or chromosomal *hfq::3xFLAG*) using an anti-FLAG antibody. RNA was purified, converted to cDNA, and subjected to high-throughput sequencing. (B) Growth curve of different *K. pneumoniae* strains (wild-type with [WT] or without [WT*] plasmids pKpn4 and pKpn6; chromosomal mutant *hfq::3xFLAG*; chromosomal mutant Δ*hfq*) grown in LB medium. (C) Expression analysis of Hfq-3xFLAG and conserved Hfq-dependent sRNAs. WT and mutant cells (*hfq::3xFLAG* and Δ*hfq*) were grown in LB. Total protein and total RNA samples were collected at indicated optical densities (0.25 to 2 and 3h after cells reached $OD_{600}$ of 2) and subjected to analysis via Western blotting (WB; upper panel) and Northern blotting (NB; lower panel), respectively. RNAP and 5S rRNA served as loading controls.

## Supplementary Figure S3

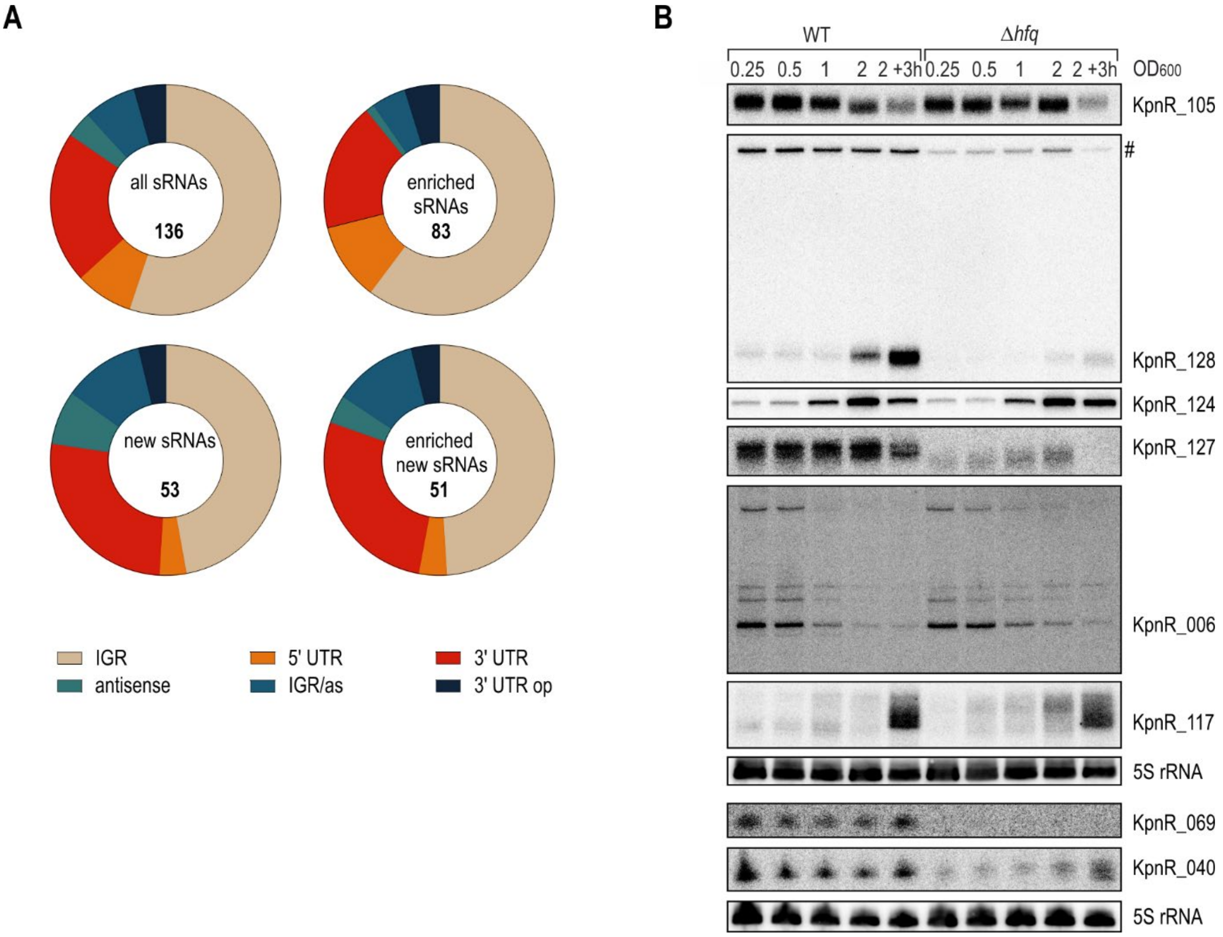


**RIP-seq analysis of Hfq-bound sRNAs and expression profiles of selected sRNA candidates.** (A) Relative distribution of different sRNA types annotated in *K. pneumoniae* (left), and distribution of different sRNA types enriched ≥3-fold in the co-IP sample compared to the WT control (right). sRNAs are categorized by their genomic location (IGR: free-standing sRNA gene in intergenic region; 5' UTR: processed from mRNA 5' end; 3' UTR: processed from mRNA 3' end; antisense: antisense to an annotated gene; IGR/as: free-standing sRNA gene in intergenic region extending into an annotated gene; 3' UTR op: free-standing sRNA gene overlapping the 3' end of an annotated gene). (B) Total RNA samples of WT and Δ*hfq* cells were collected at indicated time-points ($OD_{600}$ of 0.25 to 2 and 3h after cells reached $OD_{600}$ of 2) during growth in LB and subjected to Northern blot analysis. KpnR_128 is expressed from its own promoter internal to another transcriptional unit positioned upstream. Both transcripts share the same termination site and the ~300 nt transcript (#) is thus detected by the Kpn_R128 probe. 5S rRNA served as loading control.

## Supplementary Figure S4

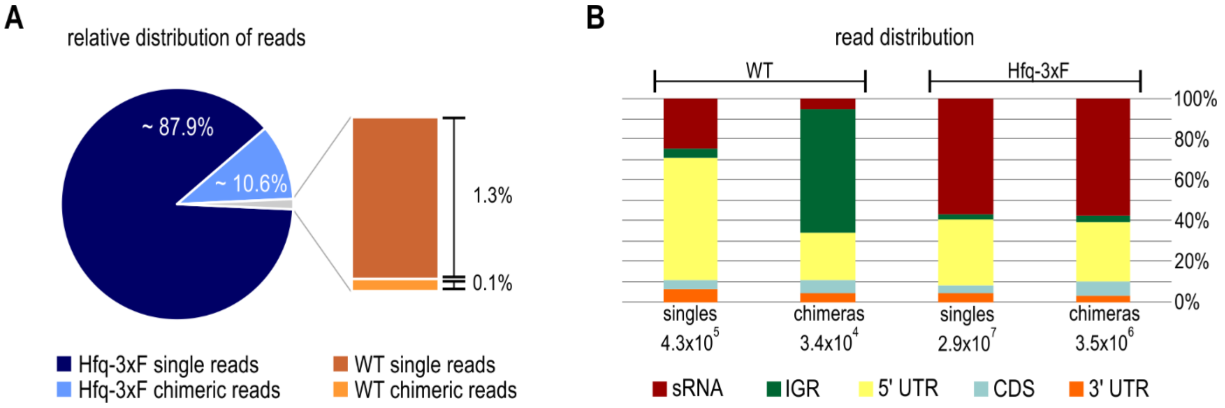


**Relative read distribution in the RIL-seq experiment.** (A) Distribution of filtered single and chimeric reads in samples recovered from the RIL-seq experiment in *K. pneumoniae* wild-type (WT) or *hfq::3xFLAG* (Hfq-3xF) cells. (B) Distribution of different RNA classes in single and chimeric reads in samples recovered from the RIL-seq experiment in *K. pneumoniae* wild-type or *hfq::3xFLAG* cells.

## Supplementary Figure S5

### A

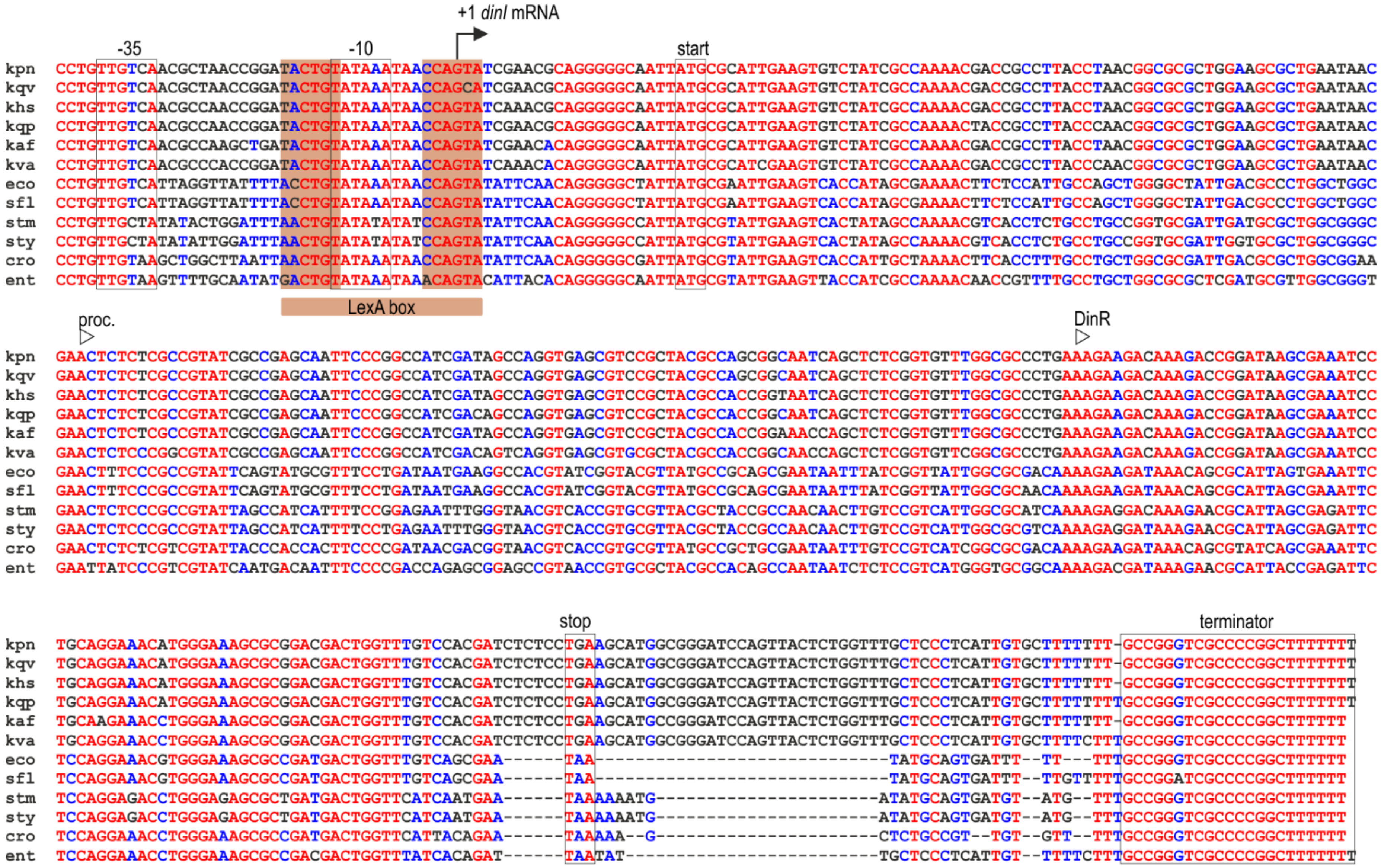

**B**

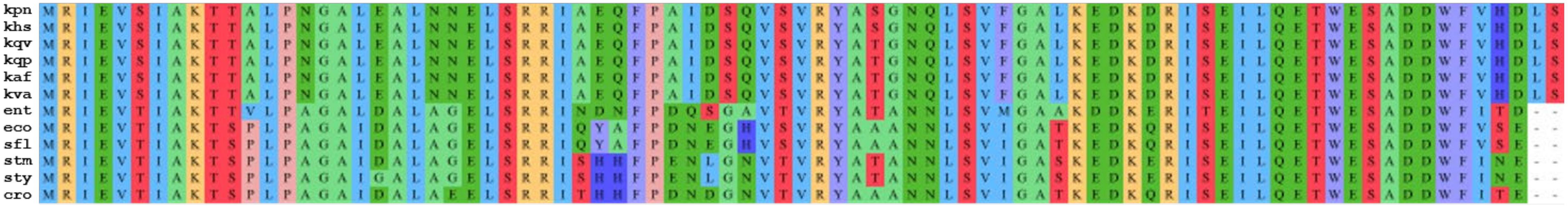


**Conservation of *dinI* in enterobacteria.** (A) Multiple alignment of the *dinI* gene of different enterobacterial species (kpn: *Klebsiella pneumoniae* MGH 78578; kqv: *Klebsiella quasivariicola* 08A119; khs: *Klebsiella pneumoniae subsp. pneumoniae* HS11286; kqp: *Klebsiella quasipneumoniae* KqPF26; kaf: *Klebsiella africana* 200023; kva: *Klebsiella variicola* LEMB11; eco: *Escherichia coli* MG1655; sfl: *Shigella flexneri* 301; stm: *Salmonella* Typhimurium LT2; sty: *Salmonella typhi* CT18; cro: *Citrobacter rodentium* ICC168; ent: *Enterobacter sp.* 638) was calculated using the MultAlin tool (2). Nucleotides are colored regarding their degree of conservation (red: high conservation; blue: partial conservation; black: little or no conservation). The -10 and -35 elements of the *dinI* promoter, as well as the start and stop codons and the Rho-independent terminator are boxed, the transcriptional start site (as determined for *K. pneumoniae*; compare Fig. S6) is indicated by an arrow. The 5’ ends of the two most abundant processing intermediates (proc. and DinR) from *dinI* mRNA in *K. pneumoniae* MGH 78578 are indicated by open triangles. The LexA box of *dinI* (as determined for *E. coli* in (3, 4)) is highlighted in orange. (B) Multiple alignment of the DinI protein of different enterobacterial species (as in (A)) was calculated using the MAFFT tool (5).

## Supplementary Figure S6

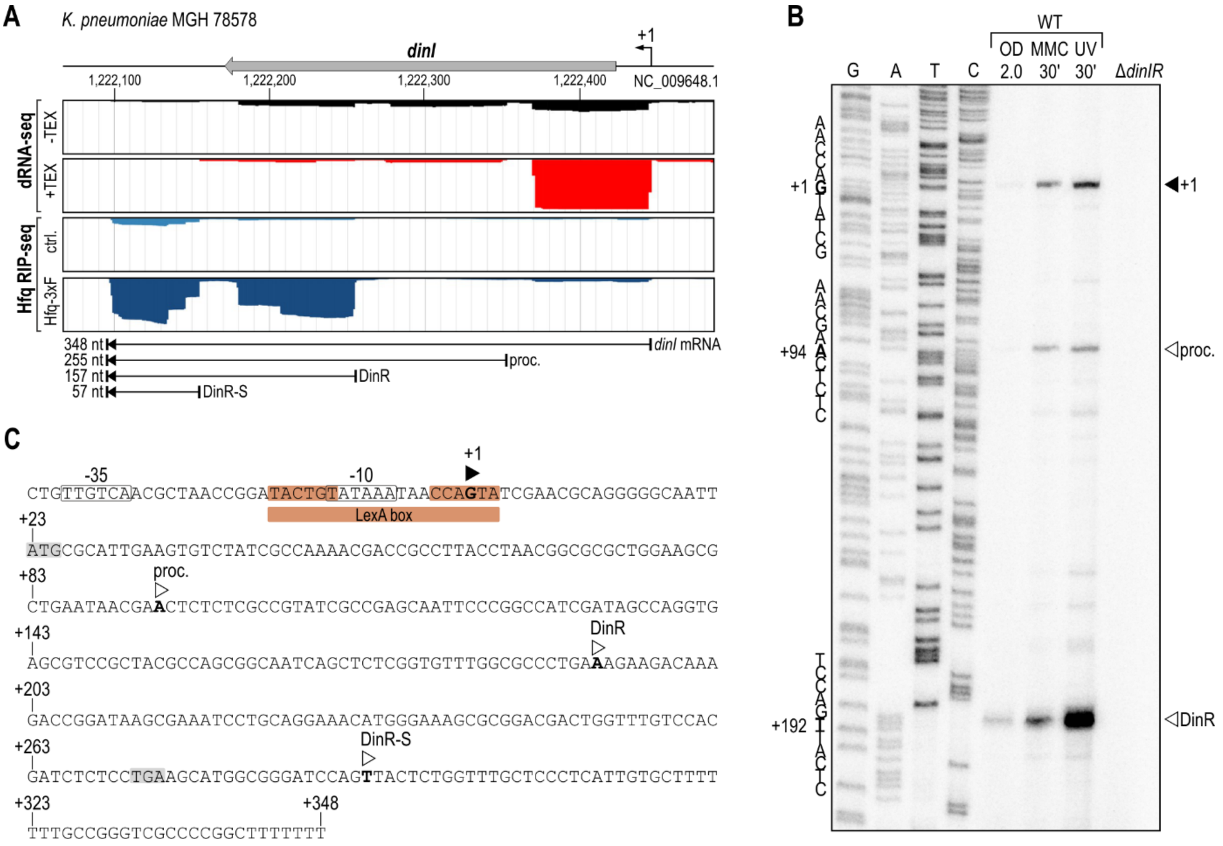


**Determination of the 5’ ends of *dinI* mRNA and its major processing products.** (A) Read-mappings of dRNA-seq (-TEX: black traces; +TEX: red traces) and Hfq RIP-seq (control: light blue traces; Hfq-3xFLAG: dark blue traces) to the *dinI* locus. The y-axes in individual experiments were set to the same scale. The positions on the *K. pneumoniae MGH 78578* genome are indicated above the sequencing traces, and the *dinI* CDS is indicated by a grey arrow. The TSS of *dinI* was determined by analysis of the dRNA-seq dataset, and is marked by a black arrow. Stable processing products were identified from dRNA-seq and Hfq RIP-seq and sizes are below the panels. (B) Primer extension analysis of *dinI* mRNA (using a gene-specific primer). RNA was extracted from wild-type cells at $OD_{600}$ of 2.0, and 30 min after DNA damage was induced through mitomycin C (MMC) or ultraviolet light (UV). RNA purified from *dinIR* mutant cells served as a control. The 5’ ends of *dinI* mRNA (black arrowhead) and its processing products (proc. and DinR; open arrowheads; DinR-S is too close to the 3’ end to be resolved in this experiment) were mapped using a sequencing ladder, and correspond to the sites determined by dRNA-seq. (C) Sequence at the *dinI* locus of *K. pneumoniae MGH 78578*. The -35 and -10 elements of the *dinI* promoter are boxed, the LexA box is high-lighted in orange. The 5’ ends of *dinI* mRNA (black arrowhead) and its processing products (proc., DinR, DinR-S; open arrowheads) are indicated. Numbering refers to the TSS as position +1.

**Supplementary Figure S7**

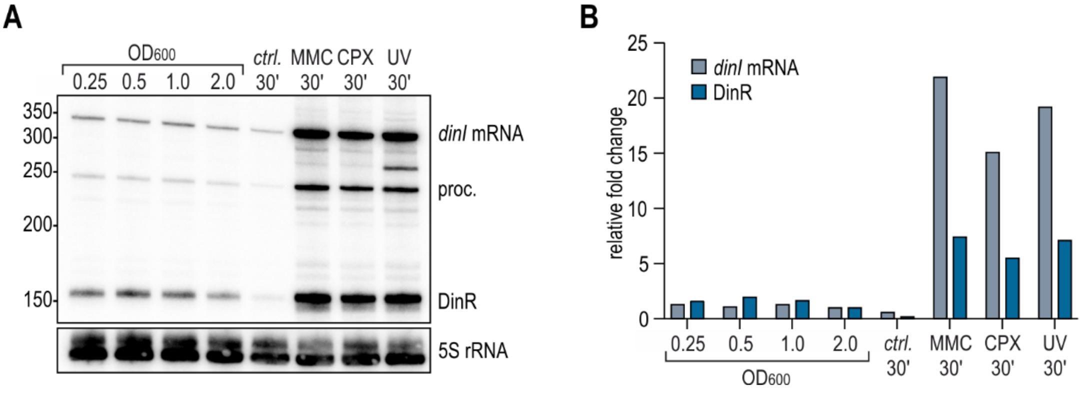


**Expression of *dinI* mRNA and DinR sRNA in *K. pneumoniae*.** (A) RNA samples were collected at different time-points over growth ($OD_{600}$ from 0.25 to 2.0), and 30 min after cells had reached an $OD_{600}$ of 2.0 in the absence (*ctrl.*) or presence of DNA damage induced through mitomycin C (MMC), ciprofloxacin (CPX) or ultraviolet light (UV). Expression of *dinI* mRNA and DinR was assessed by Northern blot analysis; 5S rRNA served as loading control. (B) Quantification of *dinI* mRNA and DinR sRNA signals relative to the expression at $OD_{600}$ of 2.0.

## Supplementary Figure S8

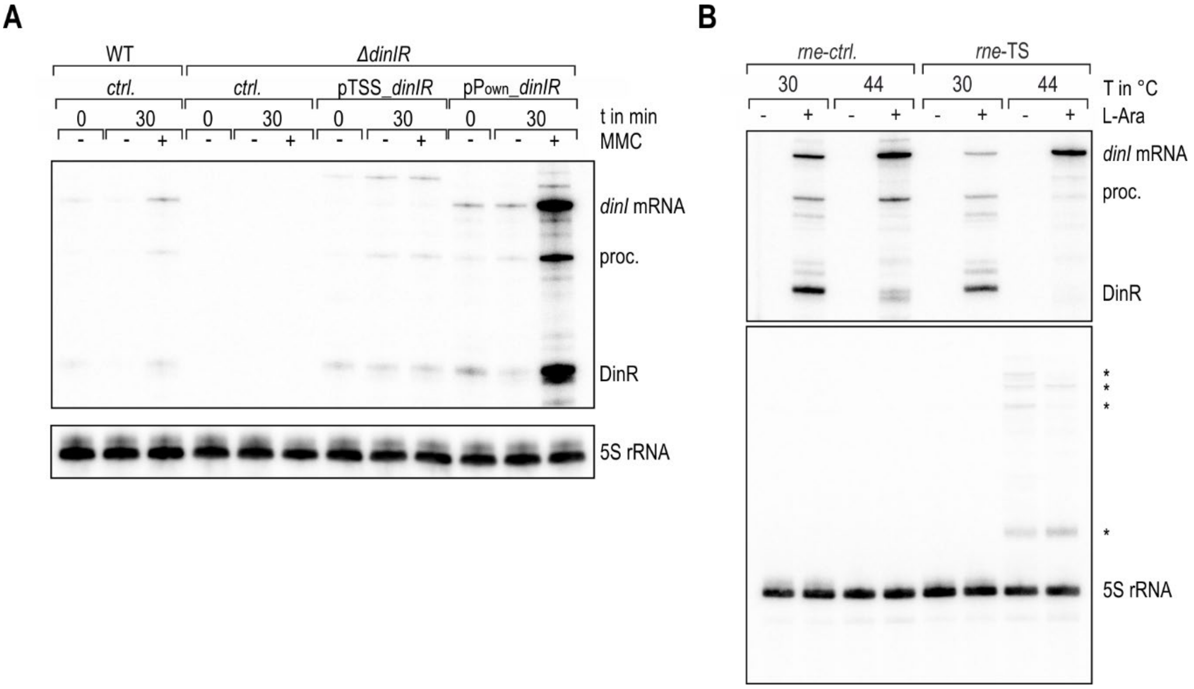


**DinR is processed from the *dinI* mRNA.** (A) *K. pneumoniae* WT or Δ*dinIR* cells carrying either an empty control vector (pBAD$_{KP}$-*ctrl.*) or plasmids harbouring different variants of the *dinI* gene (either starting from the TSS [pTSS_*dinIR*] or 50 bp upstream of the TSS [pP$_{own}$_*dinIR*]) were grown to OD$_{600}$ of 2.0, when DNA damage was induced by the addition of MMC. DinR and *dinI* mRNA levels were determined by Northern blot analysis of RNA samples collected at the indicated time-points. 5S ribosomal RNA served as a loading control. (B) *Salmonella* Typhimurium expressing a temperature-sensitive RNase E variant (*rne*-TS) or an isogenic control strain (*rne-ctrl.*) were transformed with pBAD$_{EC}$-*dinIR* and grown to OD$_{600}$ of 0.25 at the non-permissive temperature of 30 °C. Cultures were split and cultivation was continued for 45 min at 30 °C or at 44 °C to inactivate RNase E. RNA samples were collected prior to (-) and 15 min after (+) induction of *dinIR* expression with arabinose. Expression of *dinI* mRNA and DinR were determined by Northern blot analysis. 5S ribosomal RNA served as a loading control, and accumulating precursors of 5S rRNA are indicated by asterisks.

## Supplementary Figure S9

**A**

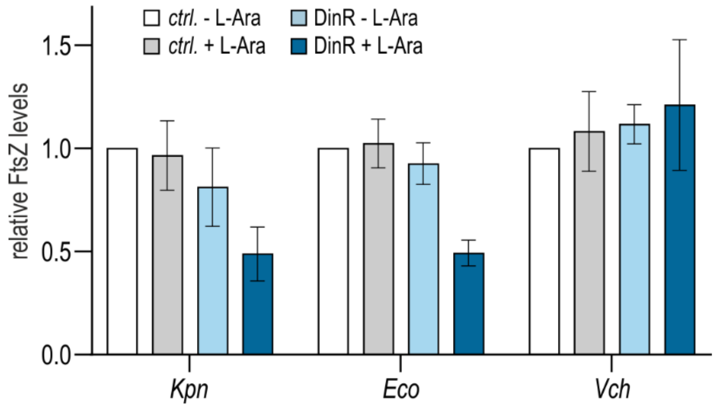


**B**

```
kpn AAAAGAGTTTTAATTTTTTAAGAGAACGCAGACAATTAGCGTTCTCAG-GCGACAGGCACAAGACGGAGAGAAACTATG
cko AAAAGAGTTTTAATTTTT-ATGAGACCGGCGACTATTAACGGTCTCAG-GCGACAGGCACAAAACGGAGAGAAACTATG
eco AAAAGAGTTTTAATTTTT-ATGAGGCCGACGATGATTA-CGGCCTCAG-GCGACAGGCACAAATCGGAGAGAAACTATG
sfl AAAAGAGTTTTAATTTTT-ATGAGGCCGACGATGATTA-CGGACTCAG-GCGACAGGCACAAATCGGAGAGAAACTATG
stm AAAAGAGTTTTAATTTTT-ATGAGGCCGCCAGATATTA-CGGCCTCAG-GCGACAGGCACATGACGGAGAGAGATTATG
vch AAAAGAGTTTTAA------------------------------C-CTGAGAGACAGGAAA--AACGGAGATAACACATG
```

**Regulation of *ftsZ* expression and conservation of the 5' UTR.** (A) Quantification of FtsZ protein levels. FtsZ expression was determined by Western blot analysis using a FtsZ-specific antiserum in total protein samples collected from *K. pneumoniae*, *E. coli* and *V. cholerae* carrying either an empty control vector ($pBAD_{KP}$-*ctrl.*, $pBAD_{EC}$-*ctrl.*, or $pBAD_{VC}$-*ctrl.*, respectively) or pBAD variants for the expression of DinR ($pBAD_{KP}$-DinR, $pBAD_{EC}$-DinR, or $pBAD_{VC}$-DinR, respectively). Bacteria were diluted from overnight cultures into fresh medium, and sRNA expression was induced by the addition of arabinose for 5 hours. FtsZ levels in the control sample (- L-Ara) was set to 1; error bars denote the standard deviation from five biological replicates. (B) Non-redundant alignment of the *ftsZ* 5' UTR of different bacterial species (kpn: *Klebsiella pneumoniae* MGH 78578; cko: *Citrobacter koseri* ATCC BAA-895; eco: *Escherichia coli* MG1655; sfl: *Shigella flexneri* 301; stm: *Salmonella* Typhimurium LT2; vch: *Vibrio cholerae* O1 El Tor N16961) was calculated using the Multalign tool (2). The stop codons of the upstream gene *ftsA* and the start codons of *ftsZ* are underlined, two transcriptional start sites (as determined by dRNA-seq in *K. pneumoniae*) are indicated by arrows. All nucleotides are colored with regard to their degree of conservation (red: high conservation; blue: partial conservation; black: little or no conservation).

**Supplementary Figure S10**

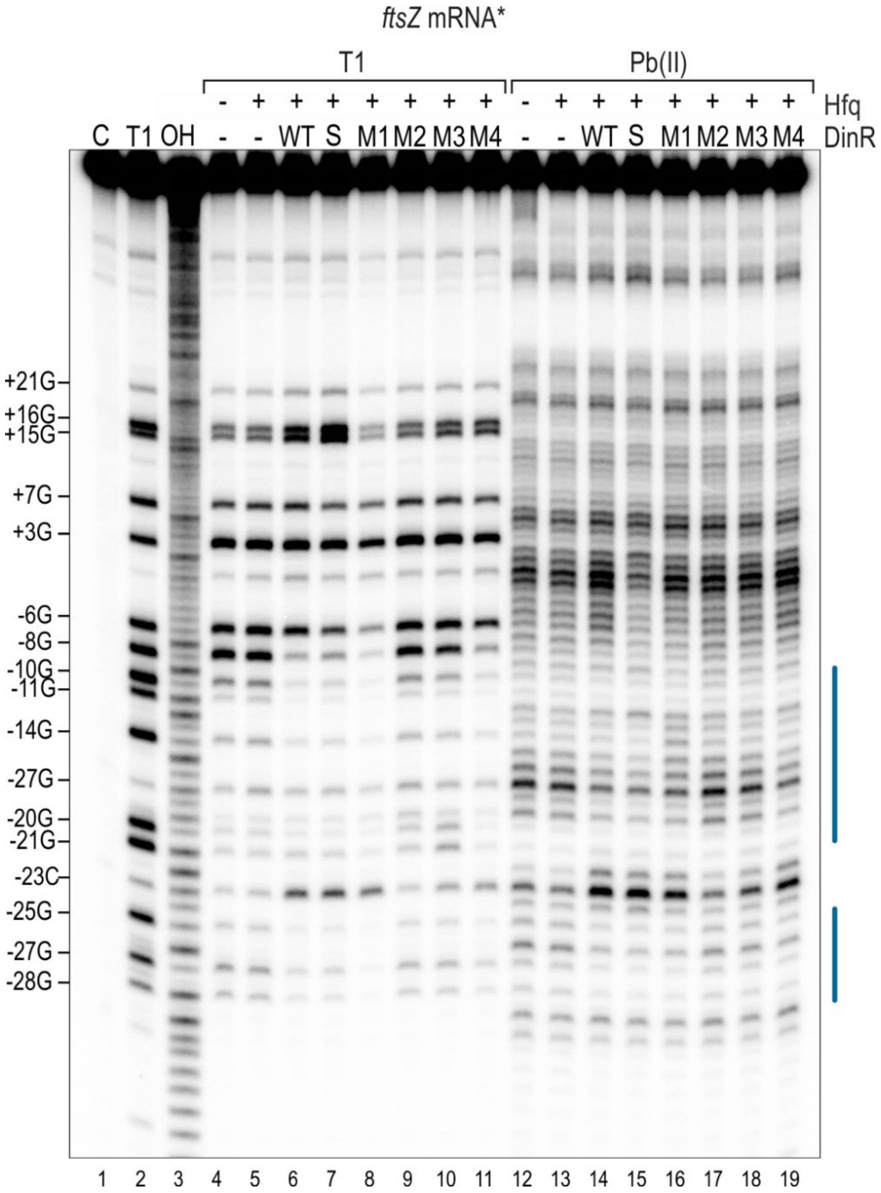


***In vitro* analysis of DinR base-pairing on *ftsZ* mRNA.** Structure probing of 5′ end-labelled *ftsZ* mRNA (TSS at –58 to +60 relative to the start codon; 0.4 pmol) with RNase T1 (lanes 4 to 11) and lead(II) acetate (lanes 12 to 19) in the absence or presence of 2 pmol (5x) Hfq protein and 4 pmol (10x) sRNAs DinR (WT), DinR-S (S), or DinR variants (M1-M4; compare 5(B)). RNase T1 and alkaline ladders of the *ftsZ* transcript were used to map the positions of individual nucleotides. The positions of G residues are indicated relative to the translational start site. Putative DinR binding sites are marked in blue.

## Supplementary Figure S11

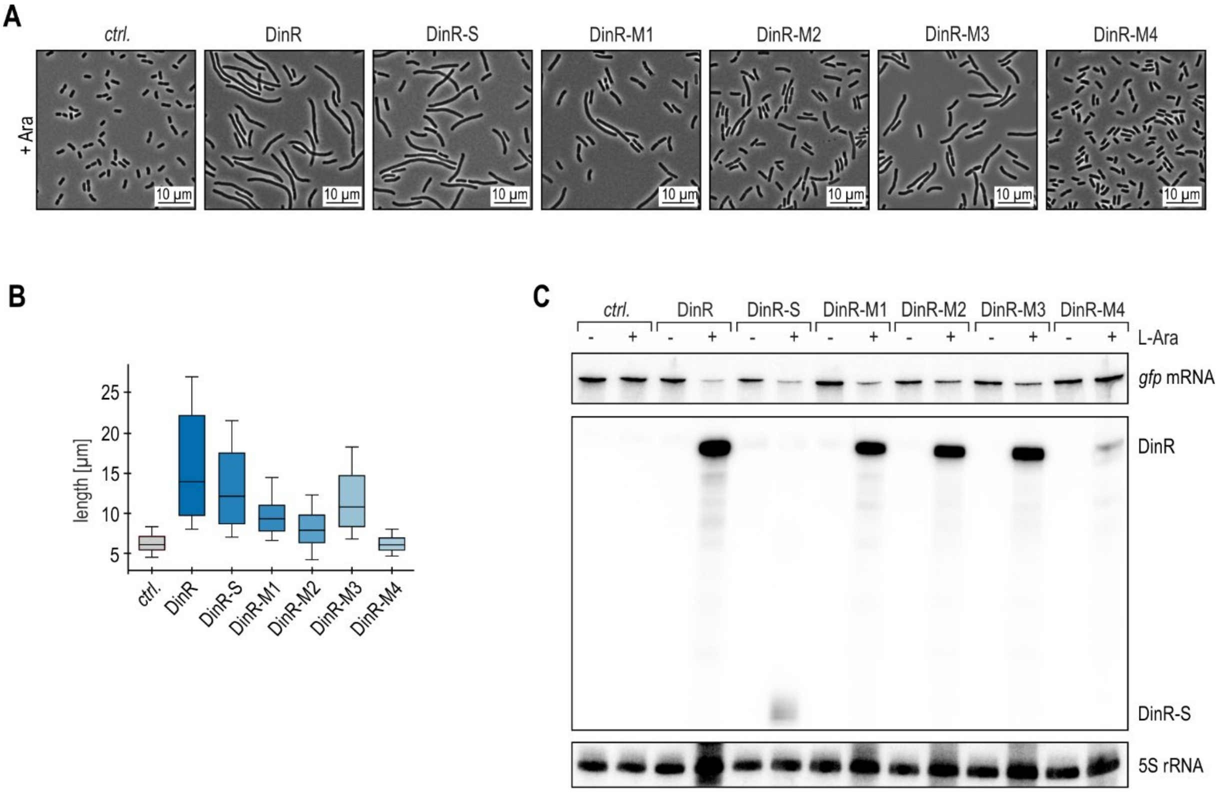


**DinR overexpression causes cell filamentation.** (A) *K. pneumoniae* carrying either an empty control vector ($pBAD_{KP}$-*ctrl.*) or $pBAD_{KP}$ variants for the expression of DinR, DinR-S, DinR-M1, DinR-M2, DinR-M3 or DinR-M4 were diluted from overnight cultures into fresh medium, and sRNA expression was induced by the addition of arabinose. Cell morphology was assessed by phase contrast microscopy after 5 hours. (B) Analysis of cell lengths in samples described in (A). The center line indicates the median, boxes represent the 25th and 75th percentiles, and lower and upper whiskers represent the 10th and 90th percentiles, respectively. (C) *E. coli* carrying the post-transcriptional *ftsZ::gfp* reporter and either $pBAD_{EC}$-*ctrl.* or $pBAD_{EC}$ variants for the expression of DinR, DinR-S, DinR-M1, DinR-M2, DinR-M3 or DinR-M4 were grown to $OD_{600}$ of 0.5. Total RNA samples were prepared from cells collected prior to (-) or 10 min after the addition of arabinose (+). DinR and *ftsZ::gfp* mRNA levels were determined by Northern blot analysis; 5S ribosomal RNA served as a loading control.

## Supplementary Figure S12

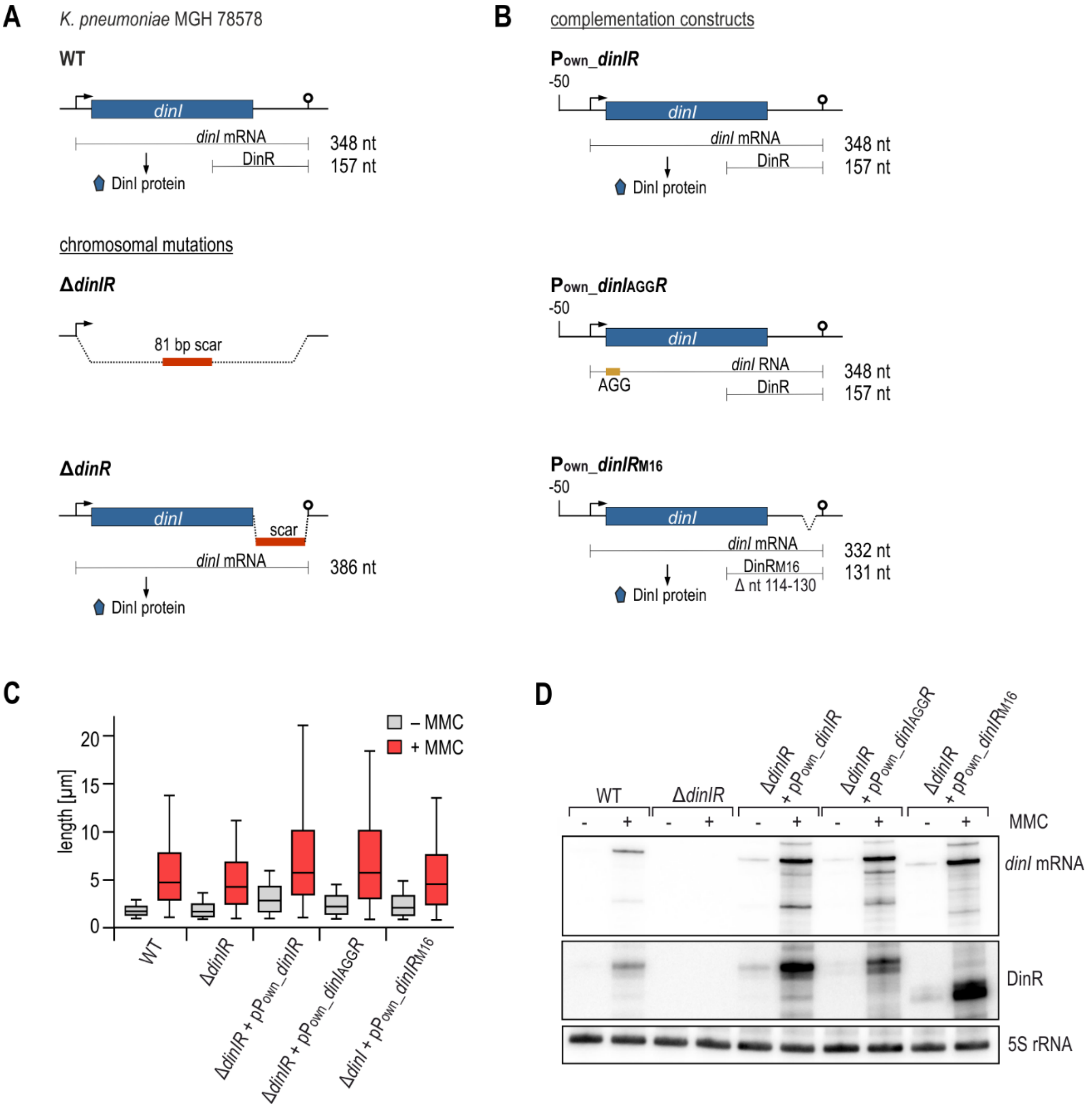


**Complementation of the *dinIR* mutant.** (A) Schematic of the *dinIR* locus of *K. pneumoniae* in wild-type and mutant cells. In the chromosomal mutant Δ*dinIR* deletion, a 81 bp scar replaces the sequence from the TSS to the end of the 3' UTR. In the chromosomal mutant Δ*dinR*, a 81 bp scar replaces the sequence from the stop codon to the beginning of the terminator hairpin (nt 84 to 126 of the sRNA). The size of the *dinI* mRNA increases to 386 nt. (B) Schematic of complementation constructs of *dinIR*. Plasmid pPown_*dinIR* expresses *dinIR* under control of its own promoter; DinI protein is produced. Plasmid pPown_*dinI*AGG*R* expresses *dinIR* under control of its own promoter; the stop codon of *dinI* is changed from ATG to AGG; DinI protein is not produced. Plasmid pPown_*dinIR*M16 expresses *dinIR* under control of its own promoter; nt 114 to 130 of DinR have been deleted; DinI protein is produced. (C) *K. pneumoniae* wild-type cells or *dinIR* mutant cells carrying complementation plasmids (as described in (B)) were diluted from overnight cultures into fresh medium, split and either treated with MMC (1 µg/mL) to induce DNA damage or left untreated. Cell morphology was assessed by phase contrast microscopy after 5 hours and quantified. The center line indicates the median, boxes represent the 25th and 75th percentiles, and lower and upper whiskers represent the 10th and 90th percentiles, respectively. (D) RNA samples prepared from cells treated as described in (C) were analyzed on Northern blot; 5S ribosomal RNA served as a loading control.

## SUPPORTING METHODOLOGY

### Construction of Bacterial Strains

*Klebsiella pneumoniae subsp. pneumoniae* MGH 78578, *E. coli* Top10 and *V. cholerae* C6706 are referred to as wild-type strains in this study.

Plasmids were introduced in *K. pneumoniae* and *E. coli* by electrotransformation, and into *V. cholerae* by conjugation via the *E. coli* S17 helper strain using standard protocols. To construct chromosomal mutations, we followed a published procedure based on the widely-used *E. coli* one-step inactivation method based on the λ-Red recombinase system (6). Briefly, wild-type cells carrying pACBSR-Hyg (encoding the λ-Red genes under control of the *araBAD* promoter) were transformed with PCR products (obtained by overlap extension PCR) containing the *aac(3)IV* gene (conferring apramycin resistance) flanked by FRT recombination sites and ~500 bp of sequences homologous to the genomic integration sites. For fragments carrying the *aac(3)IV* marker gene, pIJ773 (KFO-1407/KFO-1376) or pER15 (KFO-1375/KFO-1376) were amplified and combined with individual flanking regions (Δ*hfq*: KFO-1252/KFO-1408 and KFO-1255/KFO-1378; *hfq*-3xFLAG: KFO-1252/KFO-1377 and KFO-1255/KFO-1378; Δ*sulA*: KFO-1798/KFO-1800 and KFO-1799/KFO-1801; Δ*dinI*: KFO-1792/KFO-1821 and KFO-1905/KFO-1793; Δ*dinR*: KFO-1792/KFO-2001 and KFO-2000/KFO-1793). Transformants were recovered in the presence of apramycin, and desired mutations were confirmed by colony PCR and sequencing. The temperature-sensitive pACBSR-hyg was lost during growth in LB at 37 °C. To eliminate the *aac(3)IV* cassette, the resulting strain was transformed with pFLP-hyg, and expression of the FLP recombinase was induced with arabinose. Recombination at the FRT site and correct excision of the cassette was confirmed by colony PCR and sequencing, and the helper plasmid was removed by passaging cells at 37 °C. Multiple mutations were introduced by sequentially performing the genomic modifications described above.

Unexpectedly, introduction of the pACBSR-Hyg helper plasmid resulted in selective loss of accessory plasmids pKPN4 and pKPN6, respectively, from a subset of transformed wild-type *K. pneumoniae* MGH 78578, a phenotype that had not been reported in the original protocol. We thus verified that neither loss of the two plasmids nor addition of the epitope to the RNA-binding protein expressed from its native promoter in MGH 78578 impaired bacterial growth under standard conditions when compared to WT cells (Fig S2B). In addition, we performed phenotypic characterization of our results throughout the manuscript using an isogenic WT strain, in which pKPN4 and pKPN6 had been likewise removed.

### Bacterial growth conditions

*K. pneumoniae, Escherichia coli* and *V. cholerae* strains were grown aerobically at 37 °C in LB broth or on LB agar. Where appropriate, media were supplemented with kanamycin (50 µg/mL), chloramphenicol (20 µg/mL), ampicillin (100 µg/mL), hygromycin (100 µg/mL), or apramycin (50 µg/mL). Expression from the araBAD promoter was induced with L-arabinose (final concentration: 0.2%). Unless stated otherwise, DNA damage was triggered in *K. pneumoniae* by adding mitomycin C (MMC; 0.5 µg/mL) or ciprofloxacin (CPX; 4 µg/mL) to the culture, or by irradiation with UV light (λ=254 nm; 10 mJ/cm$^2$).

### Plasmid construction

All plasmids and oligonucleotides used in this study are listed in Tables S4 and S5.

A template plasmid (pER15) for the chromosomal integration of the 3xFLAG epitope tag was constructed via PCR amplification of pIJ773 (KFO-1250/KFO-1251), and self-ligation was carried out as in (7).

The translational GFP reporter fusion of *ftsZ* (pFS7) was constructed via Gibson assembly (GA; NEB #E2611L) as recommended by the manufacturer using a PCR product amplified

from *K. pneumoniae* genomic DNA (KFO-1713/KFO-1714) and the linearized pXG10 vector (KPO-1702/KPO-1703). For the construction of the *ftsZ(M3)::gfp* variant (pER44), plasmid pFS07 was used as a template in PCR amplification with primer set KFO-1851/KFO-1852, and the obtained fragment was self-ligated.

For plasmids expressing DinR from the arabinose-inducible pBAD plasmid, inserts amplified from *K. pneumoniae* genomic DNA were cloned into linearized vectors via GA. For expression in *E. coli*, backbone pKP8-35 was amplified via KPO-0196/KPO-0411 and combined with an insert amplified with KFO-1718/KFO-1716. For expression in *V. cholerae*, backbone pBAD1K was amplified via KPO-0196/KPO-1397 and combined with an insert amplified with KFO-1715/KFO-2140. For expression in *K. pneumoniae*, an apramycin resistance cassette (amplified from pIJ773 with KFO-1830/KFO-1831) was introduced into pKP8-35 (linearized with KFO-1832/KFO-1833), and the obtained plasmid (pER41) was amplified via KPO-0196/KPO-0411 and combined with an insert amplified with KFO-1718/KFO-1716 by GA.

For plasmids expressing variants of DinR, pFS1 or pER41 served as a template for PCR amplification with primer pairs KFO-1992/KFO-1993 (pER66 and pER90); KFO-1892/KFO-1893 (pFS51 and pER85); KFO-1849/KFO-1850 (pER43 and pER84); KFO-1990/KFO-1991 (pER65 and pER89), and obtained fragments were self-ligated.

To verify transcriptional control of DinR expression, two variants of *dinI* lacking (KFO-2028/KFO-1716; pER82) or including the annotated promoter (KFO-2029/KFO-1716; pER83) were inserted via GA into linearized pER41 lacking the *araBAD* promoter (amplified by KFO-2027/KPO-0411). Plasmid pER83 served as template for PCR amplification with primer pairs KFO-2719/KFO-2720 to obtain pER156 and with KFO-2721/KFO-2723 to obtain pER158.

**T7 transcription and 5’ end-labelling of RNA**

RNA was *in vitro* synthesized and 5’ end-labelled as described before (8, 9). In short, DNA templates carrying the T7 promotor were amplified by PCR using the oligonucleotides listed in Table S5. *In vitro* transcription of RNA from template DNA was performed using the AmpliScribe T7-Flash transcription kit (Epicentre). RNA was dephosphorylated using calf-intestinal alkaline phosphatase (NEB), subsequently extracted with P:C:I (phenol/chloroform/isoamyl alcohol; 25:24:1) and ethanol precipitated. 5’ end labelling was achieved by incubation of dephosphorylated RNA with [32P]- γATP and polynucleotide kinase (NEB) for 1 h at 37 °C. Unincorporated nucleotides were removed using Amersham MicroSpin G-50 columns (Cytiva). Labelled RNA was then purified on a denaturing 6 % PAA / 7 M Urea gel, eluted with RNA elution buffer (0.1 M sodium acetate, 0.1 % SDS, 10 mM EDTA) at 4 °C overnight and recovered by P:C:I extraction.

**Riboprobes**

The riboprobes to detect DinR, *dinI* and *sulA* mRNAs were synthesized by T7-mediated *in vitro* transcription of ~200 ng of template DNA (amplified on *Klebsiella* gDNA with KFO-1762/KFO-1763; KFO-2554/KFO/2555; KFO-2288/KFO-2260) in the presence of [$^{32}$P]-α-UTP with the MAXIscript T7 transcription kit (Invitrogen). Unincorporated nucleotides were removed using MicroSpin G-50 columns (Cytiva).

**Primer extension analysis**

For primer extension, 5 µg of RNA were denaturated in the presence of 1 pmol 5′ end-labelled primer (KFO-1745) at 70°C for 2 min and adjacently chilled on ice for 5 min. Next, the samples were mixed with the reaction mix (1X first strand buffer, 5 mM DTT, 0.5 mM each dATP, dGTP, dCTP and dTTP) at 42°C, and SuperScript III (100 U; Invitrogen) was added. cDNA synthesis was performed at 50°C for 60 min, followed by incubation at 70°C for 15 min to inactivate the enzyme. Samples were treated with RNase H (2.5 U) for 15 min at 37°C and the reaction was

stopped by the addition of GLII loading buffer. Samples was separated by electrophoresis on a 6% sequencing gel together with a template-specific ladder (prepared using the SequiTherm EXCELII DNA Sequencing Kit).

**dRNA sequencing**

In brief, RNA samples were fragmented using ultrasound, followed by treatment with polynucleotide kinase (PNK). For the depletion of processed transcripts and the control reaction, equal amounts of RNA were incubated with or without terminator 5′-phosphate-dependent exonuclease (TEX; Lucigen). RNA samples were poly(A)-tailed using poly(A) polymerase and 5′-triphosphates were removed by applying tobacco acid pyrophosphatase (TAP). An RNA adapter was ligated to the 5' end of the RNA prior to cDNA synthesis using an oligo(dT) primer and M-MLV-RNase H- reverse transcriptase. Upon amplification of the cDNA with a high-fidelity polymerase and gel fractionation, the resulting libraries were pooled and sequenced on an Illumina NextSeq 500 system with 1x75 bp read length.
Sequencing reads from the dRNA-seq experiment were processed and analysed according to (10) with minor modifications. Briefly, for every replicate in both conditions, the coverage and based on it, the difference between neighboring nucleotides was computed. Local maxima in the differences were called as TSS if the difference between maximum and minimum coverage in a 7 nt window around the TSS was above 3 TPM, and the ratio between the maximum and the minimum was above 1.3. The set of putative TSS was manually curated and the resulting predictions were used to annotate genes with a 5' UTR within a window of 250 nt upstream of an annotated gene. The most distant TSS was then used to define the 5' UTR of the corresponding gene. 3' UTRs were annotated by extending an area downstream of a gene, up to 150 nt in length unless the next annotation was closer than that window. Custom scripts are available from GitHub (https://github.com/maltesie/KpnInteractomePaper; DOI: 10.5281/zenodo.8409716).

**Hfq RIP-seq analysis**

Demultiplexed raw reads were imported into the CLC Genomics Workbench (Qiagen) and subjected to quality control and adaptor trimming. The trimmed reads were mapped to the *Klebsiella pneumoniae subsp. pneumoniae* MGH 78578 reference genome (NCBI accession numbers NC_009648.1, NC_009649.1, NC_009651.1, and NC_009653.1) with standard parameter settings. Annotations of sRNAs were added manually. Fold enrichment in samples expressing Hfq-3xFLAG over the untagged control samples was calculated using the CLC "Differential Expression for RNA-Seq" tool.

**RIL-seq analysis**

Briefly, cells corresponding to 40 $OD_{600}$ units were subjected to UV crosslinking of proteins and RNA followed by cell lysis and co-immunoprecipitation using a monoclonal anti-FLAG antibody (Sigma; #F1804). Recovered RNA was trimmed by RNase A/T1 treatment, and proximal RNAs were ligated. Upon proteinase K treatment, RNA was extracted, fragmented and DNase digested. A previously published protocol (11) was adapted for the strain-specific depletion of ribosomal RNA. Co-immunoprecipitated RNA samples were mixed with an oligonucleotide mix (final concentrations: 5.8 nM for 16S and 23S oligos; 11.6 nM for 5S oligos) in the presence of 1x SSC and 1 mM EDTA. The mixture was denatured, cooled down and incubated with streptavidin beads (ThermoFisher; #65001) in 0.5x SSC at room temperature and 50°C for 5 min each, respectively. Depleted RNA in the supernatant was purified using AMPure XP beads (Beckman-Coulter; #A63881) and subjected to cDNA library preparation. Upon PCR amplification, libraries were sequenced in paired-end mode on an Illumina NextSeq 1000 with 150 bp read-length.

The sequence file was demultiplexed using the split_libs command from RNASeqTools (DOI: 10.5281/zenodo.8388882) and the resulting files were analyzed with ChimericFragments (DOI: 10.5281/zenodo.8376810) used with the default parameters set except for the following: min_seed_length=15, autocomplete_utrs=false, bp_shift_weight=0.0, max_bp_fdr=0.35 and min_reads=5 as described in (12).

**Annotation of sRNAs**

Complementation of sRNA annotation was based on several criteria. Conserved enterobacterial sRNAs were identified by comparison with published datasets (13-16). Candidate sRNAs in IGRs were selected if a clear TSS, a length ranging from 50 to 350 nts, and a termination site were identified; sequences were scanned for putative ORFs (≥ 20 aa).
5' UTR-derived sRNAs were annotated based on the detection of a processing site or a sharp drop in coverage upstream of the translational start site of the associated mRNA. 3' UTR-derived sRNAs were selected based on the presence of a processing site or a TSS within the 3' end of the associated mRNA and a shared termination site.

**Identification of LexA motifs**

To identify previously unknown sRNAs contributing to the regulation of the SOS response we examined the Hfq RIP-seq dataset for potentially LexA-controlled transcripts, exploiting our accurate annotation of TSSs by dRNA-seq. We used MEME (17) to compute a LexA position weight matrix based on the sequences of 21 known SOS boxes of *E. coli* (3), and then searched for the obtained motif (WACTGTATATWHAHMCAGTA) within *K. pneumoniae* transcription initiation sites (-70 to +30 relative to the TSS). We identified putative LexA binding sites preceding 71 transcription units (see Table S2).

**Chromatin immunoprecipitation (ChIP)**

ChIP was performed following the previously published procedures (4, 10) with minor modifications. In brief, *K. pneumoniae* MGH 78578 wild-type cells were grown in two biological replicates in LB medium to a final $OD_{600}$ of 2.0. The culture was split, and incubation was continued for an additional 30 min in the presence or absence of MMC (1 µg/mL).
Formaldehyde was added at a final concentration of 1% to cross-link DNA and proteins, and the reaction was quenched after 20' by the addition of glycine (0.5 M). Cells were washed in 1x TBS, and lysed in lysis buffer (50 mM Hepes-KOH pH=7.0, 150 mM NaCl, 1 mM EDTA, 1% Triton X-100, 0.1% sodium deoxycholate, 0.1% SDS) containing 4 mg/ml lysozyme. DNA was sheared to an average size of ~200 bp using a BioRuptor (5x 30 s pulses). Insoluble material was removed by centrifugation, and a sample of the supernatant was stored as input control. The samples were mixed with protein A magnetic beads (Thermo Fisher #10001D), split and either incubated with anti-LexA antibody (Sigma #06-719; +AB) or no antibody (-AB) at 4°C overnight. Samples were washed twice in lysis buffer, once in modified lysis buffer (500 mM NaCl), once in ChIP wash buffer (10 mM Tris-HCl pH=8.0, 250 mM LiCl, 1 mM EDTA, 0.5% Nonidet-P40, 0.5% sodium deoxycholate), and once in TE. Samples were eluted in elution buffer (50 mM Tris-HCl, 1 mM EDTA, 1% SDS) at 65°C, and cross-linking was reverted by boiling samples (including the input) for 10 min. DNA was purified by P:C:I extraction, and analyzed by quantitative PCR (qPCR) using the GoTaq qPCR Master Mix (Promega #A6002) on a CFX96 Real-Time PCR system (Bio-Rad), with *sgrR* CDS as a control.

**Image analysis**

The pipeline detects the bacteria and includes a background subtraction, noise removal and automated thresholding. To prevent clustering of cells in close vicinity, a second branch segments the halo around each object and subtracts those from the results of the cell

thresholding. To resolve overlaps and measure cell lengths, the segmented objects are transformed into a graph structure that contains information about the cell's medial axis and diameter. This representation allowed the usage of an algorithm to split overlapping clusters into individual objects by the removal of junction vertices and addition of alternative edges minimize the cell curvature. The length of each graph component and thus the bacterial cell is determined by a JIPipe-provided operation.
A detailed explanation of the workflow and its individual components, all results as well as the utilized software versions, is available through https://asbdata.hki-jena.de/RuhlandEtAl2023_PNAS.

## Supplementary Table S1 – sRNAs in *K. pneumoniae* MGH 78578

| Number | alternative name | reference genome | left end | right end | strand | classification | reference | enrichment RIP-seq MEP | enrichment RIP-seq ESP | pot. ORF ≥ 20 aa [a] |
|---|---|---|---|---|---|---|---|---|---|---|
| **KpnR_001** | tpke11 | NC_009648.1 | 15539 | 15614 | + | IGR | (15) | n.d. | n.d. | |
| **KpnR_002** | DapZ | NC_009648.1 | 41908 | 41977 | + | 3' UTR | (15) | 7.3 | 6.0 | |
| **KpnR_003** | SroA | NC_009648.1 | 79067 | 79132 | - | 5' UTR | (15) | 0.5 | 0.3 | |
| **KpnR_004** | SgrS | NC_009648.1 | 81037 | 81261 | + | IGR | (15) | n.d. | n.d. | |
| **KpnR_005** | KPN_RS00380_5' (sRNA 1) | NC_009648.1 | 81244 | 81448 | + | 5' UTR | (14) | 0.4 | 2.5 | |
| **KpnR_006** | pdhR_3' | NC_009648.1 | 137318 | 137597 | + | 3' UTR | this study | 0.6 | 3.2 | |
| **KpnR_007** | tp2 | NC_009648.1 | 137544 | 137673 | - | IGR | (15) | 1.8 | 7.9 | |
| **KpnR_008** | rpsB_5' | NC_009648.1 | 215558 | 215695 | + | 5' UTR | (15) | 0.3 | 0.2 | |
| **KpnR_009** | rpsB_3' | NC_009648.1 | 216411 | 216509 | + | 3' UTR | this study | 12.0 | 33.7 | |
| **KpnR_010** | SraA | NC_009648.1 | 442430 | 442486 | - | IGR | (15) | n.d. | 0.8 | |
| **KpnR_011** | 4.5S_RNA | NC_009648.1 | 461884 | 462024 | + | IGR | (15) | 0.4 | 0.2 | |
| **KpnR_012** | ChiX | NC_009648.1 | 510362 | 510442 | + | IGR | (15) | 27.5 | 15.4 | |
| **KpnR_013** | KPN_RS02895_5' (sRNA 6) | NC_009648.1 | 597429 | 597620 | + | IGR | (14) | 38.9 | 56.9 | |
| **KpnR_014** | SroC | NC_009648.1 | 769973 | 770129 | - | IGR | (15) | 12.7 | 10.3 | |
| **KpnR_015** | KPN_RS03910_3915_IGR | NC_009648.1 | 817664 | 817879 | - | IGR | this study | 10.6 | 3.6 | 33 aa |
| **KpnR_016** | SdhX | NC_009648.1 | 827990 | 828076 | + | 3' UTR | (15) | 12.5 | 20.7 | |
| **KpnR_017** | KPN_RS04185_AS | NC_009648.1 | 865729 | 865826 | + | as | this study | 1.0 | 0.3 | |
| **KpnR_018** | KPN_RS04690_4695_IGR1 | NC_009648.1 | 975650 | 975775 | - | IGR | this study | 21.1 | 10.2 | 25 aa |
| **KpnR_019** | KPN_RS04690_4695_IGR2 | NC_009648.1 | 976289 | 976417 | - | IGR | this study | 9.2 | 3.9 | |
| **KpnR_020** | RybB | NC_009648.1 | 984425 | 984505 | - | IGR | (15) | 16.6 | 6.6 | |
| **KpnR_021** | KPN_RS05160_5165_IGR | NC_009648.1 | 1089328 | 1089557 | - | IGR | this study | 9.1 | 12.4 | 39 aa |
| **KpnR_022** | KPN_RS05190_3' | NC_009648.1 | 1097112 | 1097453 | + | 3' UTR | this study | 16.4 | 4.8 | |
| **KpnR_023** | ompA_5' | NC_009648.1 | 1121359 | 1121526 | - | 5' UTR | this study | 132.2 | 12.6 | |
| **KpnR_024** | KPN_RS31110_KPN_RS31115_IGR | NC_009648.1 | 1178232 | 1178539 | + | IGR/as | this study | 11.2 | 10.4 | 33 aa |
| **KpnR_025** | phoH_5' | NC_009648.1 | 1198613 | 1198845 | + | 5' UTR | this study | 0.8 | 1.5 | |
| **KpnR_026** | RtT_RNA_1 | NC_009648.1 | 1200277 | 1200414 | - | IGR | (15) | 0.5 | 2.3 | |
| **KpnR_027** | RtT_RNA_2 | NC_009648.1 | 1205516 | 1205645 | - | IGR | (15) | 3.6 | n.d. | |
| **KpnR_028** | lpxP_3' | NC_009648.1 | 1217934 | 1218011 | - | 3' UTR | (15) | n.d. | n.d. | |
| **KpnR_029** | DinR | NC_009648.1 | 1222098 | 1222253 | - | 3' UTR | this study | 4.6 | 5.3 | |
| **KpnR_030** | SraB | NC_009648.1 | 1237475 | 1237592 | + | IGR | (15) | 3.3 | 0.3 | |
| **KpnR_031** | KPN_RS05910_3' | NC_009648.1 | 1256448 | 1256635 | + | 3' UTR | this study | n.d. | 36.1 | |
| **KpnR_032** | potD_3' | NC_009648.1 | 1283992 | 1284057 | - | 3' UTR | this study | 52.5 | 10.7 | |
| **KpnR_033** | spy_5' | NC_009648.1 | 1378348 | 1378441 | + | IGR | this study | 19.8 | 13.1 | |

| | | | | | | | | | | |
|---|---|---|---|---|---|---|---|---|---|---|
| **KpnR_035** | spy3' | NC_009648.1 | 1379048 | 1379111 | + | 3' UTR | (15) | 0.1 | 0.1 | |
| **KpnR_036** | RyhB2 | NC_009648.1 | 1404504 | 1404605 | - | as | (15) | 8.2 | 11.1 | |
| **KpnR_037** | FnrS | NC_009648.1 | 1539850 | 1539972 | + | IGR | (15) | 26.2 | 7.4 | |
| **KpnR_038** | KPN_RS07525_7530_IGR (sRNA 9) | NC_009648.1 | 1564287 | 1564584 | + | IGR | (14) | 26.7 | 9.3 | |
| **KpnR_039** | KPN_RS32550_07555_IGR (sRNA 10) | NC_009648.1 | 1566904 | 1567072 | + | IGR | (14) | 0.8 | 9.2 | |
| **KpnR_040** | KPN_RS28800_07710_IGR | NC_009648.1 | 1595047 | 1595119 | - | IGR | this study | 16.8 | 10.3 | |
| **KpnR_041** | MicC | NC_009648.1 | 1603837 | 1603953 | + | IGR | (15) | 6.1 | 42.8 | |
| **KpnR_042** | KPN_RS08035_8040_IGR (sRNA 12) | NC_009648.1 | 1668378 | 1668448 | + | IGR | (14) | 0.8 | 1.1 | |
| **KpnR_043** | fumA_3' | NC_009648.1 | 1686189 | 1686371 | + | 3' UTR | this study | 1.7 | 3.0 | |
| **KpnR_044** | KPN_RS08415_8430_IGR1 | NC_009648.1 | 1745512 | 1745772 | + | IGR | this study | 2.6 | 9.7 | 34 aa |
| **KpnR_045** | KPN_RS08415_8430_IGR2 | NC_009648.1 | 1745946 | 1746213 | + | IGR/as | this study | 9.0 | 8.7 | |
| **KpnR_046** | KPN_RS31250_31255_IGR | NC_009648.1 | 1772623 | 1772813 | - | IGR/as | this study | 1.6 | 5.0 | 34 aa |
| **KpnR_047** | MgrR | NC_009648.1 | 1792507 | 1792605 | + | IGR | (15) | 23.1 | 17.1 | |
| **KpnR_048** | marA_AS | NC_009648.1 | 1805171 | 1805502 | + | as | this study | 23.8 | 9.2 | |
| **KpnR_049** | KPN_RS09105_9110_IGR | NC_009648.1 | 1872673 | 1872791 | + | IGR | this study | 0.4 | 0.8 | |
| **KpnR_050** | RydB | NC_009648.1 | 2117929 | 2118082 | - | IGR | (15) | 0.9 | 0.9 | |
| **KpnR_051** | KPN_RS10415_3' | NC_009648.1 | 2131408 | 2131492 | + | 3' UTR op | this study | 19.1 | 8.2 | |
| **KpnR_052** | KPN_RS11045_30440_IGR (sRNA 14) | NC_009648.1 | 2246314 | 2246444 | + | IGR | (14) | 6.9 | 11.9 | |
| **KpnR_053** | KPN_RS11175_AS | NC_009648.1 | 2271954 | 2272085 | + | as | this study | 11.4 | 8.3 | |
| **KpnR_054** | KPN_RS11285_11290_IGR | NC_009648.1 | 2295887 | 2296029 | + | IGR | this study | 1.3 | 0.5 | |
| **KpnR_055** | RprA | NC_009648.1 | 2367518 | 2367625 | + | IGR | (15) | 13.8 | 12.1 | |
| **KpnR_056** | KPN_RS31415_11650_IGR | NC_009648.1 | 2370293 | 2370405 | - | IGR | this study | 24.5 | 13.3 | |
| **KpnR_057** | OppX | NC_009648.1 | 2406354 | 2406535 | - | 5' UTR | (15) | 8.3 | 5.1 | |
| **KpnR_058** | dsbB_3' | NC_009648.1 | 2537931 | 2538143 | + | 3' UTR | this study | n.d. | 12.6 | |
| **KpnR_059** | SroD | NC_009648.1 | 2543260 | 2543341 | - | 3' UTR | (15) | 1.6 | 0.0 | |
| **KpnR_060** | KPN_RS12590_3' | NC_009648.1 | 2559201 | 2559318 | + | 3' UTR | this study | 3.2 | 3.1 | |
| **KpnR_061** | RydC | NC_009648.1 | 2580742 | 2580807 | + | IGR | (15) | 5.1 | 2.2 | |
| **KpnR_062** | RyeA | NC_009648.1 | 2580911 | 2581210 | + | IGR | (15) | 1.5 | 1.0 | |
| **KpnR_063** | SdsR | NC_009648.1 | 2580978 | 2581082 | - | IGR | (15) | 6.1 | 7.1 | |
| **KpnR_064** | MicL | NC_009648.1 | 2609852 | 2610162 | - | 3' UTR op | (15) | 5.8 | n.d. | |
| **KpnR_065** | 3'ETS-leuZ | NC_009648.1 | 2632966 | 2633029 | - | 3' UTR | (15) | 9.9 | 12.5 | |
| **KpnR_066** | DsrA | NC_009648.1 | 2645420 | 2645508 | - | IGR | (15) | 38.1 | 4.5 | |
| **KpnR_067** | ompC_KPN_RS13105_IGR | NC_009648.1 | 2654265 | 2654491 | + | IGR/as | this study | 0.5 | 1.1 | |
| **KpnR_068** | JUMPstart_RNA | NC_009648.1 | 2746038 | 2746142 | - | IGR/as | (15) | 1.1 | 1.0 | |
| **KpnR_069** | KPN_RS1358_13585_IGR | NC_009648.1 | 2761160 | 2761273 | + | IGR | this study | 12.5 | 10.1 | |

| | | | | | | | | | | |
|---|---|---|---|---|---|---|---|---|---|---|
| **KpnR_070** | CyaR | NC_009648.1 | 2775703 | 2775790 | + | IGR | (15) | 28.9 | 23.6 | |
| **KpnR_071** | MicF | NC_009648.1 | 2896241 | 2896333 | + | IGR | (15) | 18.3 | 9.4 | |
| **KpnR_072** | nupC_3' | NC_009648.1 | 3017927 | 3018210 | + | 3' UTR | this study | 3.0 | 3.1 | |
| **KpnR_073** | STnc250 | NC_009648.1 | 3075394 | 3075515 | - | 3' UTR | (15) | n.d. | n.d. | |
| **KpnR_074** | SroE | NC_009648.1 | 3122822 | 3122897 | - | 3' UTR | (15) | 1.4 | 2.8 | |
| **KpnR_075** | sseA_KPN_RS15310_IGR | NC_009648.1 | 3135762 | 3135917 | - | IGR | this study | 0.6 | 0.2 | |
| **KpnR_076** | GlmY | NC_009648.1 | 3166842 | 3167027 | - | IGR | (15) | 0.3 | 0.5 | |
| **KpnR_077** | RyfD | NC_009648.1 | 3207437 | 3207578 | - | 5' UTR | (15) | 0.3 | 0.5 | |
| **KpnR_078** | RaiZ | NC_009648.1 | 3210661 | 3210819 | + | 3' UTR | (15) | 4.1 | 104.4 | |
| **KpnR_079** | tmRNA | NC_009648.1 | 3231291 | 3231653 | + | IGR | (15) | 0.2 | 0.3 | |
| **KpnR_080** | KPN_RS16030_3' | NC_009648.1 | 3290414 | 3290609 | - | 3' UTR op | this study | 164.0 | 7.5 | |
| **KpnR_081** | MicA | NC_009648.1 | 3318096 | 3318170 | + | IGR | (15) | 5.9 | 5.9 | |
| **KpnR_082** | sok_AT | NC_009648.1 | 3394403 | 3394559 | - | 5' UTR | (15) | 0.0 | 0.5 | |
| **KpnR_083** | SokX | NC_009648.1 | 3394457 | 3394512 | + | IGR | (15) | 0.3 | 0.1 | |
| **KpnR_084** | CsrB | NC_009648.1 | 3438471 | 3438838 | - | IGR | (15) | 0.5 | 0.8 | |
| **KpnR_085** | GcvB | NC_009648.1 | 3469834 | 3470041 | + | IGR | (15) | 15.5 | 6.7 | |
| **KpnR_086** | rppH_AS | NC_009648.1 | 3555242 | 3555333 | + | as | this study | 12.7 | 10.4 | |
| **KpnR_087** | OmrA | NC_009648.1 | 3563070 | 3563157 | - | IGR | (15) | 12.4 | 7.1 | |
| **KpnR_088** | OmrB | NC_009648.1 | 3563274 | 3563356 | - | IGR | (15) | 1.1 | 5.6 | |
| **KpnR_089** | ompK26_3' | NC_009648.1 | 3587787 | 3587874 | - | 3' UTR | this study | 7.8 | 4.1 | |
| **KpnR_090** | 6S_RNA | NC_009648.1 | 3665037 | 3665220 | + | IGR | (15) | 0.2 | 0.2 | |
| **KpnR_091** | SibC | NC_009648.1 | 3665896 | 3666042 | + | 3' UTR | (15) | 2.5 | 36.2 | |
| **KpnR_092** | SroG | NC_009648.1 | 3778913 | 3779064 | - | 5' UTR | (15) | 0.4 | 0.3 | |
| **KpnR_093** | alx_5' | NC_009648.1 | 3878691 | 3878875 | + | 5' UTR | (15) | 1.4 | 0.6 | |
| **KpnR_094** | RnpB | NC_009648.1 | 3896359 | 3896741 | - | IGR | (15) | 0.3 | 0.4 | |
| **KpnR_095** | SraG | NC_009648.1 | 3934702 | 3934873 | + | IGR | (15) | 0.6 | 0.0 | |
| **KpnR_096** | ArcZ | NC_009648.1 | 3973082 | 3973203 | + | IGR/as | (15) | 14.2 | 8.1 | |
| **KpnR_097** | RyhB | NC_009648.1 | 4161791 | 4161886 | - | IGR | (15) | 12.6 | 12.0 | |
| **KpnR_098** | bscO_KPN_RS31745_IGR1 (sRNA 24) | NC_009648.1 | 4269680 | 4269841 | + | IGR | (14) | 3.1 | 4.0 | |
| **KpnR_099** | bscO_KPN_RS31745_IGR2 | NC_009648.1 | 4276216 | 4276497 | + | IGR/as | this study | 2.0 | 3.5 | 28 aa |
| **KpnR_100** | RtT_RNA_3 | NC_009648.1 | 4277971 | 4278104 | - | IGR | (15) | 0.6 | 0.5 | |
| **KpnR_101** | sok_AT | NC_009648.1 | 4299285 | 4299435 | - | 5' UTR | (15) | 3.2 | 24.4 | |
| **KpnR_102** | KPN_RS21245_21250_IGR (sRNA 25) | NC_009648.1 | 4314524 | 4314846 | + | IGR/as | (14) | 1.1 | 6.1 | |
| **KpnR_103** | KPN_RS21430_waa_IGR | NC_009648.1 | 4355486 | 4355592 | - | IGR/as | this study | 1.2 | 1.5 | |
| **KpnR_104** | IstR | NC_009648.1 | 4463773 | 4463903 | - | IGR/as | (15) | 0.9 | 0.4 | |

| | | | | | | | | | | |
|---|---|---|---|---|---|---|---|---|---|---|
| **KpnR_105** | KPN_RS22175_22180_IGR | NC_009648.1 | 4503473 | 4503564 | + | IGR | this study | 0.6 | 3.1 | |
| **KpnR_106** | RbsZ | NC_009648.1 | 4553995 | 4554205 | + | 3' UTR op | (15) | n.d. | n.d. | |
| **KpnR_107** | Spot_42 | NC_009648.1 | 4571507 | 4571625 | + | IGR | (15) | 30.0 | 17.0 | |
| **KpnR_108** | CsrC | NC_009648.1 | 4572621 | 4572867 | + | IGR | (15) | 0.3 | 0.5 | |
| **KpnR_109** | GlnZ | NC_009648.1 | 4577815 | 4577914 | - | 3' UTR | (15) | 13.1 | 8.7 | |
| **KpnR_110** | CpxQ | NC_009648.1 | 4622452 | 4622509 | + | 3' UTR | (15) | 17.2 | 6.5 | |
| **KpnR_111** | OxyS | NC_009648.1 | 4655076 | 4655194 | - | IGR | (15) | 18.7 | 17.0 | |
| **KpnR_112** | GlmZ | NC_009648.1 | 4707421 | 4707597 | + | IGR | (15) | 6.6 | 6.2 | |
| **KpnR_113** | Pseudomonas_P26 | NC_009648.1 | 4769689 | 4769817 | + | 3' UTR op | (15) | 0.0 | 0.2 | |
| **KpnR_114** | SroH | NC_009648.1 | 4782709 | 4782823 | - | IGR | (15) | 11.0 | 4.0 | |
| **KpnR_115** | aceK-int | NC_009648.1 | 4810817 | 4810901 | + | IGR | (15) | 3.1 | 4.1 | |
| **KpnR_116** | malM_3' | NC_009648.1 | 4844452 | 4844548 | + | 3' UTR op | (15) | 0.0 | 10.1 | |
| **KpnR_117** | pspG_KPN_RS23890_IGR | NC_009648.1 | 4854487 | 4854571 | + | IGR | this study | 77.7 | 8.6 | |
| **KpnR_118** | KPN_RS23935_23940_IGR (sRNA 27) | NC_009648.1 | 4866884 | 4867205 | + | IGR | (14) | 36.3 | 12.4 | |
| **KpnR_119** | KPN_RS23955_KPN_RS23965_IGR (sRNA 28) | NC_009648.1 | 4872137 | 4872383 | + | IGR | (14) | 13.6 | 5.6 | |
| **KpnR_120** | SraL | NC_009648.1 | 4885978 | 4886125 | - | IGR | (15) | 0.7 | 0.6 | |
| **KpnR_121** | iolD_3' | NC_009648.1 | 5113801 | 5113867 | + | 3' UTR | this study | 43.1 | 92.2 | |
| **KpnR_122** | iolE_3' | NC_009648.1 | 5116601 | 5116790 | + | 3' UTR | this study | 10.7 | 16.6 | |
| **KpnR_123** | repA_tap_IGR | NC_009649.1 | 5598 | 5684 | - | IGR | this study | 0.1 | 0.4 | |
| **KpnR_124** | KPN_RS30785_KPN_RS26475_IGR | NC_009649.1 | 50982 | 51224 | - | IGR | this study | 3.3 | 6.5 | |
| **KpnR_125** | pcoE_3' | NC_009649.1 | 71349 | 71428 | - | 3' UTR | this study | 32.8 | 15.5 | |
| **KpnR_126** | KPN_RS26860_KPN_RS29675_IGR | NC_009649.1 | 124376 | 124473 | - | IGR | this study | 0.0 | 0.0 | |
| **KpnR_127** | KPN_RS32575_KPN_RS26920_IGR1 | NC_009649.1 | 134020 | 134087 | - | IGR | this study | 9.1 | 7.0 | |
| **KpnR_128** | KPN_RS32575_KPN_RS26920_IGR2 | NC_009649.1 | 134372 | 134491 | + | IGR | this study | 6.0 | 5.3 | |
| **KpnR_129** | repA_tap_IGR | NC_009650.1 | 5597 | 5684 | - | IGR | this study | n.d. | n.d. | |
| **KpnR_130** | KPN_RS27500_27505_IGR | NC_009650.1 | 58478 | 58578 | - | IGR | this study | n.d. | n.d. | |
| **KpnR_131** | KPN_RS27570_27575_IGR | NC_009650.1 | 68576 | 68685 | - | IGR | this study | n.d. | n.d. | |
| **KpnR_132** | traX_KPN_RS27770_IGR | NC_009650.1 | 106802 | 106912 | - | IGR | this study | n.d. | n.d. | |
| **KpnR_133** | KPN_RS27800_27805_IGR | NC_009651.1 | 3977 | 4078 | + | IGR | this study | 0.3 | 0.3 | |
| **KpnR_134** | KPN_RS28310_KPN_RS28295_IGR | NC_009652.1 | 3747 | 3848 | - | IGR | this study | n.d. | n.d. | |
| **KpnR_135** | FadZ | NC_009648.1 | 4747195 | 4747235 | - | 3' UTR | (13) | n.d. | n.d. | |
| **KpnR_136** | MalH | NC_009648.1 | 4836639 | 4836835 | - | 3' UTR | (16) | n.d. | n.d. | |
| **KpnR_137** | ZbiJ | NC_009648.1 | 929321 | 929471 | - | 3' UTR | (13) | n.d. | n.d. | |

(a) determined for newly annotated sRNA candidates

## Supplementary Table S2 – LexA boxes in MGH 78578

| reference genome | TSS position | strand | associated gene | alternative name | motif | *E. coli* LexA regulon | Hfq-RIPseq enrichment MEP | Hfq-RIPseq enrichment ESP |
|---|---|---|---|---|---|---|---|---|
| **NC_009648.1** | 69500 | - | KPN_RS00315 | *dinA/polB* | ACCTGTATAAAACCCCAGCG | yes | 0,5 | 0,8 |
| **NC_009648.1** | 134256 | + | as in KPN_RS00615 | | TATTGTAGGTCCACTCAGGA | na | na | na |
| **NC_009648.1** | 279304 | + | KPN_RS01315 | *dinB* | TGCTGTATGGGTATACAGTG | yes | 0,6 | 0,9 |
| **NC_009648.1** | 302334 | + | KPN_RS01425 | | TAATTTTTGCACAACCAGCG | no homologue in MG1655 | 1,0 | 0,5 |
| **NC_009648.1** | 304001 | + | KPN_RS01430 | | AAATGTTCAATAAATCAGTA | no homologue in MG1655 | 1,6 | 0,8 |
| **NC_009648.1** | 495194 | - | KPN_RS02385 | *priC* | TAATGGTTAAAATAACAGGT | no | 0,5 | 0,5 |
| **NC_009648.1** | 513937 | + | KPN_RS02475 | *cueR* | TCCTGAATAATTTTCCGGTC | no | 0,4 | 0,6 |
| **NC_009648.1** | 898954 | + | KPN_RS04345 | *uvrB* | CACTGTTTAAATATCCAGTA | yes | 0,5 | 0,6 |
| **NC_009648.1** | 926126 | + | KPN_RS04480 | *dinG* | TAGTGGCTGTTTATACAGTA | yes | 0,4 | 0,6 |
| **NC_009648.1** | 938906 | - | KPN_RS04540 | *rhtA* | CCCTGTTTTTTTCAACATTA | no | 0,4 | 0,8 |
| **NC_009648.1** | 1032191 | + | KPN_RS04965 | *ftsK* | TCCTGTTAATCCATACAGCA | yes | 0,4 | 0,4 |
| **NC_009648.1** | 1122285 | - | KPN_RS05300 | *sulA* | TACTGTATATGCATACAGTA | yes | 2,8 | 2,7 |
| **NC_009648.1** | 1222445 | - | KPN_RS32185 | *dinI* | TACTGTATAAATAACCAGTA | yes | **15,2** | **7,9** |
| **NC_009648.1** | 1330706 | - | KPN_RS06320 | | CACTGTACATTAATACAGTA | no homologue in MG1655 | 0,7 | 0,7 |
| **NC_009648.1** | 1330925 | + | KPN_RS06325 | *ltrA* | TACTGTATATATATTCAGTG | no homologue in MG1655 | 0,5 | **5,7** |
| **NC_009648.1** | 1392082 | - | KPN_RS06630 | *ydjM* | TACTGTATGAATCGACAGTT | yes | 0,8 | 2,1 |
| **NC_009648.1** | 1392082 | - | KPN_RS06630 | *ydjM* | CACTGTATAAAAACCCTATA | yes | 0,8 | 2,1 |
| **NC_009648.1** | 1580772 | + | KPN_RS07645 | | TTCTGATTGTAATCACAGGA | no homologue in MG1655 | 1,9 | 1,6 |
| **NC_009648.1** | 1602046 | - | KPN_RS07740 | *uspF* | GAAGGGATACTTAAACAGGA | no | 1,5 | 0,5 |
| **NC_009648.1** | 1755352 | - | KPN_RS08465 | *dmsD* | CAATGGCTACACCACCAGCG | no | 0,7 | 1,9 |
| **NC_009648.1** | 2113961 | - | KPN_RS32425 | | TACTGTTCTTATAATCAATA | no homologue in MG1655 | 0,7 | 1,1 |
| **NC_009648.1** | 2116422 | - | KPN_RS10330 | | TACTGTATAAAAAAACAGTA | no homologue in MG1655 | 0,5 | 0,8 |
| **NC_009648.1** | 2196973 | + | internal in mdtK | *mdtK* | TACTGGTCAACATTCCGGTG | no | 2,1 | 2,7 |
| **NC_009648.1** | 2366135 | + | KPN_RS11635 | *ydiK* | TATTGATATTATTATCAGTA | no | 0,7 | 0,6 |
| **NC_009648.1** | 2445265 | - | KPN_RS12010 | *chaC* | AGCTGGATAATATTTCAGCA | no | 0,6 | 0,9 |
| **NC_009648.1** | 2548585 | - | KPN_RS12535 | *dinG* | CACTGTCCAAATAACCAGGG | yes | 0,4 | 0,6 |
| **NC_009648.1** | 2556314 | - | KPN_RS12575 | *yoaE* | TTCCGTTTAATTACTCAGGA | no | 0,6 | 0,5 |
| **NC_009648.1** | 2602370 | - | KPN_RS12815 | *ruvA* | GGCTGGATATCTATCCAGCC | yes | 0,4 | 0,4 |
| **NC_009648.1** | 2619131 | - | KPN_RS12900 | *otsB* | CACTGTCTATACTTACATGG | yes | 0,6 | 1,0 |
| **NC_009648.1** | 2689997 | - | KPN_RS13305 | *sbmC* | AGCTGTATATTCATACAGTA | yes | 0,5 | 0,4 |
| **NC_009648.1** | 2714275 | - | KPN_RS13395 | | TACTGGTTTTACTAACCGCT | no homologue in MG1655 | 1,5 | 1,3 |
| **NC_009648.1** | 2714275 | - | KPN_RS13395 | | TAATGGTTTTATACACTGGA | no homologue in MG1655 | 1,5 | 1,3 |
| **NC_009648.1** | 2803617 | + | KPN_RS13750 | | CACTTTTTAAAAATCCAGTG | no | 1,1 | **6,7** |
| **NC_009648.1** | 2867663 | - | as in KPN_RS14035 | | ATCTGGACAATAAAAAAGTT | na | na | na |
| **NC_009648.1** | 3167027 | - | KPN_RS15455 | *yfhK/glrK* | TAATGTCATATATATCAGTA | no | 0,8 | 1,2 |
| **NC_009648.1** | 3327869 | - | KPN_RS16255 | *recA* | TACTGTATGACCATACAGTA | yes | 0,6 | 0,4 |
| **NC_009648.1** | 3350324 | + | KPN_RS16375 | | AACTGGTATTTTATCCAGTA | yes | 0,5 | 0,5 |
| **NC_009648.1** | 3350324 | + | KPN_RS16375 | | ACCTGTTTACAATAACTGGT | yes | 0,5 | 0,5 |
| **NC_009648.1** | 3646387 | - | KPN_RS17835 | *yqfA* | CACTGGAATCATTCTCAGTT | no | 0,5 | 0,9 |
| **NC_009648.1** | 3803003 | - | KPN_RS18625 | *mug* | TGCTGTTTTTTATAAACAATG | no | 0,4 | 0,6 |
| **NC_009648.1** | 3829108 | - | KPN_RS30150 | | TACTGTATGTTTATACAGTA | no homologue in MG1655 | 1,4 | 1,2 |
| **NC_009648.1** | 3893961 | + | KPN_RS19135 | *yhaK* | ATCTGGCTAACAATACAGCG | no | **11,7** | 1,3 |
| **NC_009648.1** | 3904868 | - | KPN_RS19195 | *agaR* | AAATGAAAGTTATTCCAGGA | no | 1,0 | 1,3 |
| **NC_009648.1** | 4068128 | - | KPN_RS20110 | *rpsJ* | TGATGATTTTCTAAACAGCA | no | 1,0 | 1,9 |
| **NC_009648.1** | 4113170 | + | int in KPN_RS20365 | | TTCTGATATTCCTTACAGCT | na | na | na |
| **NC_009648.1** | 4121558 | - | KPN_RS20400 | *ompR* | AAATGTATACTTAACCTGCT | no | 0,5 | 0,6 |
| **NC_009648.1** | 4170640 | - | KPN_RS20610 | *phd antitoxin* | TGTTGTACAATAAAAGAGTA | no | na | na |
| **NC_009648.1** | 4182892 | + | KPN_RS20675 | *ydcR* | TGTCGTTTATTTACACAGGA | no | na | na |
| **NC_009648.1** | 4275970 | - | KPN_RS21060 | *dppA* | CGCTGGATGTAAAACCCGGC | no | na | na |

| | | | | | | | | |
|---|---|---|---|---|---|---|---|---|
| **NC_009648.1** | 4378809 | + | KPN_RS21565 | *rpoZ* | GACTGAACCCACATTCAGTA | no | 0,7 | 0,9 |
| **NC_009648.1** | 4416113 | - | KPN_RS21735 | *nlpA* | AGATGATTTTATTTCCAGTT | no | 1,1 | 1,9 |
| **NC_009648.1** | 4463983 | + | KPN_RS21985 | *tisB* | TACTGTTCATCCATACAGTG | yes | 1,3 | **4,3** |
| **NC_009648.1** | 4508691 | + | KPN_RS22205 | | TGCTGTATAATTAACCAGTT | no homologue in MG1655 | 2,2 | 1,7 |
| **NC_009648.1** | 4585490 | - | KPN_RS22560 | *aes* | CACTGGATATTAAAACAGTA | no | 0,8 | 1,3 |
| **NC_009648.1** | 4718603 | + | KPN_RS23230 | *uvrD* | CTCTGTATATATTAACAGGG | yes | 0,8 | 1,7 |
| **NC_009648.1** | 4726754 | - | as in KPN_RS23265 | *rhtC* | ATCTGGTAGCCCATCCAGCA | na | na | na |
| **NC_009648.1** | 4732717 | - | KPN_RS23295 | *metR* | TACTGTATATTCCTCAAGCG | no | 0,8 | 0,9 |
| **NC_009648.1** | 4737198 | + | KPN_RS23315 | *rmuC* | AACTGGACGTTTGTACAGCA | yes | 0,6 | 0,5 |
| **NC_009648.1** | 4764396 | + | KPN_RS23455 | *tRNA-Gly* | TAATGGCTATTACCTCAGCC | no | na | na |
| **NC_009648.1** | 4849108 | + | KPN_RS23845 | *lexA* | AACTGTATATACACCCAGGG | yes | 1,1 | **3,4** |
| **NC_009648.1** | 4849108 | + | KPN_RS23845 | *lexA* | AGCTGTATATACTCACAGCA | yes | 1,1 | **3,4** |
| **NC_009648.1** | 4864335 | - | KPN_RS23925 | *uvrA* | TACTGTATATTCATTCAGGT | yes | 0,8 | 0,5 |
| **NC_009648.1** | 4864417 | + | KPN_RS23930 | *ssb1* | ACCTGAATGAATATACAGTA | yes | 0,6 | 0,5 |
| **NC_009648.1** | 5137712 | - | KPN_RS30735 | | TGCTGTATAAATATACATTA | no homologue in MG1655 | 2,4 | 1,1 |
| **NC_009648.1** | 5137763 | + | as in KPN_RS25310 | | TAATGTATATTTATACAGCA | na | na | na |
| **NC_009648.1** | 5308814 | - | KPN_RS26165 | *robA* | CACTGAATGCTAAAACATCA | no | 0,7 | 0,8 |
| **NC_009649.1** | 109182 | + | KPN_RS26790 | | ACCTGTATCCATAAACAGTG | no homologue in MG1655 | 1,0 | 1,2 |
| **NC_009650.1** | 29810 | - | KPN_RS27330 | | CGCTGTAAAACAAAACAGGC | no homologue in MG1655 | na | na |
| **NC_009650.1** | 50040 | - | KPN_RS27430 | *umuD* | TACTGTATGCATAACCAGTA | yes | na | na |
| **NC_009651.1** | 61448 | - | KPN_RS28135 | *umuD* | TACTGTATGCATAACCAGTA | yes | 0,7 | 0,9 |
| **NC_009653.1** | 525 | + | orphan | | TGATGTTTAAACTTATAGTG | na | na | na |

## Supplementary Table S3 – Bacterial strains

| strain | stock name | genotype/relevant markers | | source/reference |
|---|---|---|---|---|
| ***Klebsiella pneumoniae subsp. pneumoniae MGH 78578*** | | | | |
| **WT** | KFKS-031 | pKPN3-7 | 1; 2; 3A/C/D; 4; 6A/B; S1; S2B/C; S3A; S4; S6A; S7; S8A; S9A; S11A/B; S12C/D | (14) |
| **WT** | KFKS-245 | pKPN3; pKPN5; pKPN7 | 3E; 6C; S2B; S3B; S6B | this study |
| **Δ*hfq*** | KFKS-024 | pKPN3; pKPN5; pKPN7; Δ*hfq* | 3C/E; S2B/C; S3B | this study |
| ***hfq::3xFLAG*** | KFKS-061 | pKPN3; pKPN5; pKPN7; *hfq::3xFLAG* | 1; 2; 3A; 4A; S2B/C; S3A; S4; S6A | this study |
| **Δ*dinIR*** | KFKS-198 | pKPN3; pKPN5; pKPN7; Δ*dinI* | 6; S6B; S8A; S12C/D | this study |
| **Δ*dinR*** | KFKS-199 | pKPN3; pKPN5; pKPN7; Δ*dinR* | 6 | this study |
| **Δ*sulA*** | KFKS-230 | pKPN3; pKPN5; pKPN7; Δ*sulA* | 6 | this study |
| **Δ*dinIR* Δ*sulA*** | KFKS-231 | pKPN3; pKPN5; pKPN7; Δ*dinI* Δ*sulA* | 6 | this study |
| **Δ*dinR* Δ*sulA*** | KFKS-232 | pKPN3; pKPN5; pKPN7; Δ*dinR* Δ*sulA* | 6 | this study |
| ***Escherichia coli*** | | | | |
| **WT** | KFS-0088 | TOP10<br>*F- mcrA Δ(mrr-hsdRMS-mcrBC) φ80lacZΔM15 ΔlacX74 nupG recA1 araD139 Δ(ara-leu)7697 galE15 galK16 rpsL(StrR) endA1 λ-* | 4B/C/D; 5D/E; S9A; S11C | Invitrogen |
| | KFS-0033 | S17<br>*ΔlacU169 (ΦlacZΔM15) recA1 endA1 hsdR17 thi-1 gyrA96 relA1 λpir* | | New England Biolabs |
| ***Vibrio cholerae* O1 El Tor C6706** | | | | |
| **WT** | KPS-0014 | | 4B/C/D; S9A | (18) |
| ***Salmonella* Typhimurium SL1344** | | | | |
| ***rne*-ctrl.** | JVS-6999 | *[rluC-rne]IG::cat* | S6B | (19) |
| ***rne*-TS** | JVS-7000 | *[rluC-rne]IG::cat rne-3071 (ts)* | S6B | (19) |

## Supplementary Table S4 – Plasmids

| trivial name | plasmid ID | description | origin, marker | used in Figure | reference |
|---|---|---|---|---|---|
| **pBAD$_{EC}$-*ctrl.*** | pKP8-35 | empty vector (used in *E. coli*) | pBR322, Amp$^R$ | 4B/C/D; 5D/E; S9A; S11C | (9) |
| **pXG10** | pXG10-sfGFP | empty vector (used in *E. coli*) | pSC101*, Cm$^R$ | | (9) |
| **pBAD$_{KP}$-*ctrl.*** | pER41 | empty vector (used in *K. pneumoniae*) | pBR322, Apm$^R$ | 4B/C/D/E; S8A; S9A; S11A/B | this study |
| **pBAD$_{VC}$-*ctrl.*** | pBAD1K-ctrl | empty vector (used in *V. cholerae*) | p15A, Kan$^R$ | 4B/C/D; S9A | (20) |
| **pACBSR-hyg** | | temperature-sensitive plasmid to express λRED-recombinase system from the arabinose-inducible *araBAD* promoter | p15A, Hyg$^R$ | | (6) |
| **pFLP-hyg** | | temperature-sensitive Flp recombinase expression plasmid | p15A, Hyg$^R$ | | (6) |
| **pIJ773** | | template for apramycin resistance (*aac(3)IV*) and flanking FRT sites to construct chromosomal deletions in *K. pneumoniae* | pBR322, Apm$^R$ | | (6) |
| **pIJ773-3xFLAG** | pER15 | template for apramycin resistance (*aac(3)IV*) and flanking FRT sites to construct chromosomal *K. pneumoniae hfq::3xFLAG* | pBR322, Apm$^R$ | | this study |
| **pBAD$_{EC}$-DinR** | pFS1 | expression of DinR under control of the *araBAD* promoter in *E. coli* | pBR322, Amp$^R$ | 4B/C/D; 5D/E; S9A; S11C | this study |
| **pBAD$_{KP}$-DinR** | pER45 | expression of DinR under control of the *araBAD* promoter in *K. pneumoniae* | pBR322, Apm$^R$ | 4B/C/D/E; S9A; S11A/B | this study |
| **pBAD$_{VC}$-DinR** | pER114 | expression of DinR under control of the *araBAD* promoter in *V. cholerae* | p15A, Kan$^R$ | 4B/C/D; S9A | this study |
| ***ftsZ::gfp*** | pFS7 | expression of *ftsZ::gfp* translational fusion (-62 to /60 of *ftsZ* relative to the translational start site) under control of the $P_{LtetO-1}$ promoter | pSC101*, Cm$^R$ | 5D/E | this study |
| **pBAD$_{EC}$-DinR-M3** | pER43 | expression of DinR-M3 under control of the *araBAD* promoter in *E. coli* | pBR322, Amp$^R$ | 5D/E; S11C | this study |
| **pBAD$_{KP}$-DinR-M3** | pER84 | expression of DinR-M3 under control of the *araBAD* promoter in *K. pneumoniae* | pBR322, Apm$^R$ | S11A/B | this study |
| ***ftsZ*-M3*::gfp*** | pER44 | expression of *ftsZ-M3::gfp* translational fusion (-62 to /60 of *ftsZ* relative to the translational start site) under control of the $P_{LtetO-1}$ promoter | pSC101*, Cm$^R$ | 5D/E | this study |
| **pBAD$_{EC}$-DinR-M2** | pFS51 | expression of DinR-M2 under control of the *araBAD* promoter in *E. coli* | pBR322, Amp$^R$ | S11C | this study |
| **pBAD$_{KP}$-DinR-M2** | pER85 | expression of DinR-M2 under control of the *araBAD* promoter in *K. pneumoniae* | pBR322, Apm$^R$ | S11A/B | this study |
| **pBAD$_{EC}$-DinR-M4** | pER65 | expression of DinR-M4 under control of the *araBAD* promoter in *E. coli* | pBR322, Amp$^R$ | S11C | this study |
| **pBAD$_{KP}$-DinR-M4** | pER89 | expression of DinR-M4 under control of the *araBAD* promoter in *K. pneumoniae* | pBR322, Apm$^R$ | S11A/B | this study |
| **pBAD$_{EC}$-DinR-M1** | pER66 | expression of DinR-M1 under control of the *araBAD* promoter in *E. coli* | pBR322, Amp$^R$ | S11C | this study |
| **pBAD$_{KP}$-DinR-M1** | pER90 | expression of DinR-M1 under control of the *araBAD* promoter in *K. pneumoniae* | pBR322, Apm$^R$ | S11A/B | this study |
| **pBAD$_{EC}$-DinRshort** | pFS54 | expression of DinRshort under control of the *araBAD* promoter in *E. coli* | pBR322, Amp$^R$ | S11C | this study |
| **pBAD$_{KP}$-DinRshort** | pER95 | expression of DinRshort under control of the *araBAD* promoter in *K. pneumoniae* | pBR322, Apm$^R$ | 4B/C/D; 5D/E; S9A; S11C | this study |
| **pBAD$_{EC}$-*dinIR*** | pER37 | expression of *dinIR* under control of the *araBAD* promoter in *E. coli* | pBR322, Amp$^R$ | S11A/B | this study |
| **pTSS_*dinIR*** | pER82 | expression of *dinIR* transcript from TSS (-22 relative to the translational start site) in *K. pneumoniae* | pBR322, Apm$^R$ | S8B | this study |
| **pPown_*dinIR*** | pER83 | expression of *dinI*R transcript -47 from TSS (-69 relative to the translational start site) in *K. pneumoniae*; mutation of *dinI* start codon (ATG to AGG) | pBR322, Apm$^R$ | S8A | this study |
| **pPown_*dinI*$_{AGG}$*R*** | pER156 | expression of *dinI*R transcript -47 from TSS (-69 relative to the translational start site) in *K. pneumoniae* | pBR322, Apm$^R$ | S8A; S12C/D | this study |
| **pPown_*dinIR*$_{M16}$** | pER158 | expression of *dinI*R transcript -47 from TSS (-69 relative to the translational start site) in *K. pneumoniae;* DinR lacks nt 114-130 | pBR322, Apm$^R$ | S12C/D | this study |

## Supplementary Table S5 – Oligonucleotides

| oligo ID | Sequence 5‘ to 3‘ | description |
|---|---|---|
| **oligonucleotides for construction of plasmids** | | |
| **KFO-1250** | GATCATGACATCGATTACAAGGATGACGATGACAAGTAGTAACTGG AGCTGCTTCGAAGTTC | plasmid construction pER15 |
| **KFO-1251** | GATGTCATGATCTTTATAATCACCGTCATGGTCTTTGTAGTCCCTAC ACATCGAATTCCTGC | plasmid construction pER15 |
| **KPO-1702** | ATGCATGTGCTCAGTATCTCTATC | linearization pXG10 |
| **KPO-1703** | GCTAGCGGATCCGCTGG | linearization pXG10 |
| **KPO-0196** | GGAGAAACAGTAGAGAGTTGCG | linearization pBAD/pBAD1K |
| **KPO-0411** | TTTTTCTAGATTAAATCAGAACGC | linearization pBAD; construction pER82/pER83 |
| **KPO-1397** | GATCCGGTGATTGATTGAGC | linearization pBAD1K |
| **KFO-1713** | GAGATACTGAGCACATGCATTTTTTTAAGAGAACGCAGACAATTAG | plasmid construction pFS7 |
| **KFO-1714** | GAGCCAGCGGATCCGCTAGCGCCACCGACGCCGATG | plasmid construction pFS7 |
| **KPO-1715** | CAACTCTCTACTGTTTCTCCAAGAAGACAAAGACCGGATAAG | plasmid construction pFS1/ER114 |
| **KPO-1716** | TCTGATTTAATCTAGAAAAACGATATTGAGCAGAATAATCAGG | plasmid construction pFS1/pER37/pER82/pER83 |
| **KFO-1832** | ACTCTTCCTTTTTCAATATTATTGAAG | plasmid construction pER41 |
| **KFO-1833** | CTGTCAGACCAAGTTTACTC | plasmid construction pER41 |
| **KFO-1830** | AATATTGAAAAAGGAAGAGTATGTCATCAGCGGTGGAG | plasmid construction pER41 |
| **KFO-1831** | GAGTAAACTTGGTCTGACAGTCAGCCAATCGACTGGCG | plasmid construction pER41 |
| **KFO-1787** | CAACTCTCTACTGTTTCTCCGTATCGAACGCAGGGGG | plasmid construction pER37 |
| **KFO-2027** | GGTAACGAATCAGACAATTGAC | plasmid construction ER82/pER83 |
| **KFO-2028** | GTCAATTGTCTGATTCGTTACCGTATCGAACGCAGGGGG | plasmid construction pER82 |
| **KFO-2029** | GTCAATTGTCTGATTCGTTACCCGCAGCCCCCTGTTG | plasmid construction pER83 |
| **KFO-2140** | GCTCAATCAATCACCGGATCCGATATTGAGCAGAATAATCAGG | plasmid construction pER114 |
| **oligonucleotides for mutagenesis** | | |
| **KFO-1849** | CATTGTGGTTTTTTTGCCGGGTCGCC | plasmid construction pER43 |
| **KFO-1850** | CAAAAAAACCACAATGAGGGAGCAAACC | plasmid construction pER43 |
| **KFO-1851** | CGACAGCCACAAGACGGAGAGAAACTATG | plasmid construction pER44 |
| **KFO-1852** | CTTGTGGCTGTCGCCTGAGAACGC | plasmid construction pER44 |
| **KFO-1892** | CCTCATTCTGCTTTTTTTGCCGGGTCG | plasmid construction pFS51 |
| **KFO-1893** | AAAAGCAGAATGAGGGAGCAAACCAGAG | plasmid construction pFS51 |
| **KFO-1898** | CGCAACTCTCTACTGTTTCTCCTTACTCTGGTTTGCTCCCTC | plasmid construction pFS54 |
| **KPO-0196** | GGAGAAACAGTAGAGAGTTGCG | plasmid construction pFS54 |
| **KFO-1990** | CTTTTTTTCCCGGGTCGCCCCGGC | plasmid construction pER65 |
| **KFO-1991** | CCCGGGAAAAAAAGCACAATGAGG | plasmid construction pER65 |
| **KFO-1992** | GTTTGCTGCCTCATTGTGCTTTTTTTG | plasmid construction pER66 |
| **KFO-1993** | AATGAGGCAGCAAACCAGAGTAACTG | plasmid construction pER66 |
| **KFO-2719** | GCAATTAGGCGCATTGAAGTGTCTATCG | plasmid construction pER156 |
| **KFO-2720** | CAATGCGCCTAATTGCCCCCTGCGTTCG | plasmid construction pER156 |
| **KFO-2721** | TTTTTGCCGGGTCGCCC | plasmid construction pER158 |
| **KFO-2723** | GGGCGACCCGGCAAAAACAAACCAGAGTAACTGGATCC | plasmid construction pER158 |
| **oligonucleotides for Northern blot probing** | | |
| **KPO-0009** | CTACGGCGTTTCACTTCTGAGTTC | oligoprobe 5S rRNA |
| **KPO-0353** | GAATTGGGACAACTCCAGTG | oligoprobe *gfp* |
| **KFO-0510** | GAATACTGCGCCAACACCAG | oligoprobe ArcZ |
| **KFO-0748** | GCCCGTCAAAGAGGAATTTC | oligoprobe ChiX |
| **KFO-1297** | CTCGTTCCGGCTCAGGA | oligoprobe GcvB |
| **KFO-1745** | GGCAAAAAAAGCACAATGAGGGAGCAAACCAG | oligoprobe DinR |
| **KFO-1853** | AGTTTCTCTCCGTCTTGTGC | oligoprobe *ftsZ* |
| **KFO-1973** | AATGAGGGAGCAAACCAGAGTAA | oligoprobe DinR |
| **KFO-2239** | AAGCAGGCGAACATCACTGG | oligoprobe KpnR_128 |
| **KFO-2394** | GTACGGGCAATCATCATATTGG | oligoprobe MicL |
| **KFO-2401** | CTAAGGAGGTGGTTCCTGG | oligoprobe CyaR |
| **KFO-2423** | CGAAGATCAGATCTAAAGTTTGG | oligoprobe KpnR_105 |
| **KFO-2551** | GTTCGGCTGTATGTAGGGTAC | oligoprobe KpnR_117 |
| **oligonucleotides for strain construction** | | |
| **KFO-1407** | TGTAGGCTGGAGCTGCTTC | amplification aac(3)IV::FRT |
| **KFO-1376** | CCGGGGATCCGTCGACC | amplification aac(3)IV::FRT |
| **KFO-1375** | GACTACAAAGACCATGACGG | amplification aac(3)IV::FRT::3xFLAG |
| **KFO-1252** | GATACCGCCAGATGTGGTC | Δ*hfq* and *hfq*::3xFLAG |
| **KFO-1255** | AGCGCCGGTCGCTTTAAC | Δ*hfq* and *hfq*::3xFLAG |
| **KFO-1378** | GGTCGACGGATCCCCGGGGCCAGTGCTGTTTTTCCAC | Δ*hfq* |
| **KFO-1408** | GAAGCAGCTCCAGCCTACAATTCTCTCTTTTCCTTATATGTAT | Δ*hfq* |
| **KFO-1377** | CCGTCATGGTCTTTGTAGTCTTCGGCGTCGTCGCTGTC | *hfq*-3xFLAG |
| **KFO-1378** | GGTCGACGGATCCCCGGGGCCAGTGCTGTTTTTCCAC | *hfq*-3xFLAG |
| **KFO-1798** | GGTCGGACCGTTGTTGTTC | Δ*sulA* |

| | | |
|---|---|---|
| **KFO-1799** | CAGGCCTTCAGATAAATGTTC | Δ*sulA* |
| **KFO-1800** | GGTCGACGGATCCCCGGGTGAAATTAGGATTATTCCTGG | Δ*sulA* |
| **KFO-1801** | GAAGCAGCTCCAGCCTACACATAGTCTATGCCGCCTTTAG | Δ*sulA* |
| **KFO-1814** | AATATCAAGCTTATCGATACCG | amplification pIJ773 |
| **KFO-1815** | CATCGAATTCCTGCAGCC | amplification pIJ773 |
| **KFO-1792** | GCAATGCCTGCTTCCAGG | Δ*dinI* and Δ*dinR* |
| **KFO-1793** | GCAGCATCTGATGTTCAACC | Δ*dinI* and Δ*dinR* |
| **KFO-1905** | CGAAGCAGCTCCAGCCTACATGGTTATTTATACAGTATCCGG | Δ*dinI* |
| **KFO-1821** | GCAGGTCGACGGATCCCCGGATCCCCTCTTCTGTTCAGAATAG | Δ*dinI* |
| **KFO-2000** | CGAAGCAGCTCCAGCCTACATCAGGAGAGATCGTGGAC | Δ*dinR* |
| **KFO-2001** | GCAGGTCGACGGATCCCCGGCTTTTTTTGCCGGGTCGCC | Δ*dinR* |
| **oligonucleotides for T7 templates** | | |
| **KFO-1762** | GTTTTTTTTAATACGACTCACTATAGGCGACCCGGCAAAAAAAG | fwd DinR riboprobe |
| **KFO-1763** | GATAAGCGAAATCCTGCAGG | rev DinR riboprobe |
| **KFO-2288** | GTTTTTTTTAATACGACTCACTATAGGCTGGGCAGCGTTAACGAG | fwd *sulA* riboprobe |
| **KFO-2260** | CGAATGGGTTCAGTCCGC | rev *sulA* riboprobe |
| **KFO-2554** | GTTTTTTTTAATACGACTCACTATAGGACGCTCACCTGGCTATC | fwd *dinI* riboprobe |
| **KFO-2555** | CGCATTGAAGTGTCTATCGC | rev *dinI* riboprobe |
| **KFO-1899** | GTTTTTTTTAATACGACTCACTATAGGTTACTCTGGTTTGCTCCCTC | fwd T7 template DinRshort |
| **KFO-1900** | GTTTTTTTTAATACGACTCACTATAGGAAGAAGACAAAGACCGGATA AG | fwd T7 template DinR |
| **KFO-1691** | AAAAAAAGCCGGGGCGACC | rev T7 template DinR/DinRshort |
| **KFO-1902** | GTTTTTTTTAATACGACTCACTATAGGTTAAGAGAACGCAGACAATT AG | fwd T7 template *ftsZ* |
| **KFO-1901** | GCCACCGACGCCGATG | rev T7 template *ftsZ* |
| **oligonucleotide for primer extension** | | |
| **KFO-1745** | GGCAAAAAAAGCACAATGAGGGAGCAAACCAG | primer extension *dinIR* |
| **oligonucleotides for qPCR (ChIP analysis)** | | |
| **KFO-2724** | GCGATAGACACTTCAATGCG | qPCR $P_{dinI}$ |
| **KFO-2725** | GTGAAGAAATAAAAACGCAGCC | qPCR $P_{dinI}$ |
| **KFO-2726** | GTAAAGCGTGTTCGCTTCG | qPCR $P_{recA}$ |
| **KFO-2727** | CTATGCGCTAAATCTGCTCTG | qPCR $P_{recA}$ |
| **KFO-2756** | GTATTGCCCTGCAGCGTC | qPCR *sgrR* |
| **KFO-2757** | GCTCAAGATCCATGCCTTTG | qPCR *sgrR* |
| **oligonucleotides for rRNA depletion** | | |
| **23S_1** | [Btn]ACCTTTCCCTCACGGTACTGGTTCGCTATCGGTCA | 23S rRNA depletion #1 |
| **23S_2** | [Btn]AGTCGCTGGCTCATTATACAAAAGGTACGCCGTCACC | 23S rRNA depletion #2 |
| **23S_3** | [Btn]TCGGGGAGAACCAGCTATCTCCGGGTTTGATTGGC | 23S rRNA depletion #3 |
| **23S_4** | [Btn]GTGGCTGCTTCTAAGCCAACATCCTG | 23S rRNA depletion #4 |
| **23S_5** | [Btn]GGGTACAGGAATATTAACCTGATTTCCATCGACTACGCC | 23S rRNA depletion #5 |
| **23S_6** | [Btn]CACCTGTGTCGGTTTGGGGTACGGT | 23S rRNA depletion #6 |
| **23S_7** | [Btn]TCGTGCGGGTCGGAACTTACCCGACAAG | 23S rRNA depletion #7 |
| **23S_8** | [Btn]GAGCCGACATCGAGGTGCCAAACA | 23S rRNA depletion #8 |
| **23S_9** | [Btn]CGGCGGATAGGGACCGAACTGTCTCACGAC | 23S rRNA depletion #9 |
| **16S_1** | [Btn]CCGCTCGACTTGCATGTGTTAAGCATGCCGACAGCGTTCG | 16S rRNA depletion #1 |
| **16S_2** | [Btn]CCCATTGTGCAAGATTCCCTACTGCTGCCTCCCGT | 16S rRNA depletion #2 |
| **16S_3** | [Btn]ACCGCGGCTGCTGGCACGGAGT | 16S rRNA depletion #3 |
| **16S_4** | [Btn]ACGGCGTGGACTACCAGGGTAT | 16S rRNA depletion #4 |
| **16S_5** | [Btn]TCCACATGCTCCACCGCTTGTGCGGGCCCCCG | 16S rRNA depletion #5 |
| **16S_6** | [Btn]ACCCAACATCTCACAACACGAGCTGACGACA | 16S rRNA depletion #6 |
| **16S_7** | [Btn]GGGCAGTGTGTACAAGGCCCGGGA | 16S rRNA depletion #7 |
| **16S_8** | [Btn]AAGGAGGTGATCCAGCCGCAG | 16S rRNA depletion #8 |
| **16S-gram-_1** | [Btn]CACGTCCTTCATCGCCTTTTACTGCCAAGGCATCC | 16S rRNA depletion - Gram negative bacteria #1 |
| **16S-gram-_2** | [Btn]CCACACCCGGCCTATCAACGTGGTGGTCTTCGACG | 16S rRNA depletion - Gram negative bacteria #2 |
| **5S_1** | [Btn]GTTCGGGAAGGGGTCAGGTGGGTCCAACGCGCTA | 5S rRNA depletion #1 |
| **5S_2** | [Btn]AGACCCCACACTACCATCGGCGATACGTCG | 5S rRNA depletion #2 |
| **oligonucleotides for RIL-seq** | | |
| **3Tr3** | P-AGATCGGAAGAGCACACGTCTG-ddC | ligation to the cDNA 3′ end |
| **AR2** | TACACGACGCTCTTCCGAT | reverse transcription primer |
| **BC5_adaptor** | P-AATCACTTGAGATCGGAAGAGCGTCGTGTA-ddC | ligation to the RNA 3′ end |
| **BC6_adaptor** | P-ACCAAGTCGAGATCGGAAGAGCGTCGTGTA-ddC | ligation to the RNA 3′ end |
| **BC7_adaptor** | P-ACAACTCGCAGATCGGAAGAGCGTCGTGTA-ddC | ligation to the RNA 3′ end |
| **BC8_adaptor** | P-ACCCGTCTTAGATCGGAAGAGCGTCGTGTA-ddC | ligation to the RNA 3′ end |
| **P5_Enr** | AATGATACGGCGACCACCGAGATCTACACTCTTTCCCTACACGACG CTCTTCCGATCT | PCR enrichment |
| **P7_BC1_Enr** | CAAGCAGAAGACGGCATACGAGATTCGTGTGCGTGACTGGAGTTC AGACGTGTGCTCTTCCGATCT | PCR enrichment |

## Supplementary References


1. Kim D, *et al.* (2012) Comparative analysis of regulatory elements between Escherichia coli and Klebsiella pneumoniae by genome-wide transcription start site profiling. *PLoS Genet* 8(8):e1002867.
2. Corpet F (1988) Multiple sequence alignment with hierarchical clustering. *Nucleic Acids Res* 16(22):10881-10890.
3. Fernandez De Henestrosa AR, *et al.* (2000) Identification of additional genes belonging to the LexA regulon in Escherichia coli. *Mol Microbiol* 35(6):1560-1572.
4. Wade JT, Reppas NB, Church GM, & Struhl K (2005) Genomic analysis of LexA binding reveals the permissive nature of the Escherichia coli genome and identifies unconventional target sites. *Genes Dev* 19(21):2619-2630.
5. Katoh K, Rozewicki J, & Yamada KD (2019) MAFFT online service: multiple sequence alignment, interactive sequence choice and visualization. *Brief Bioinform* 20(4):1160-1166.
6. Huang TW, *et al.* (2014) Capsule deletion via a lambda-Red knockout system perturbs biofilm formation and fimbriae expression in Klebsiella pneumoniae MGH 78578. *BMC Res Notes* 7:13.
7. Sharma CM, Darfeuille F, Plantinga TH, & Vogel J (2007) A small RNA regulates multiple ABC transporter mRNAs by targeting C/A-rich elements inside and upstream of ribosome-binding sites. *Genes Dev* 21(21):2804-2817.
8. Fröhlich KS, Papenfort K, Fekete A, & Vogel J (2013) A small RNA activates CFA synthase by isoform-specific mRNA stabilization. *EMBO J* 32(22):2963-2979.
9. Papenfort K, *et al.* (2006) $s^E$-dependent small RNAs of *Salmonella* respond to membrane stress by accelerating global *omp* mRNA decay. *Mol Microbiol* 62(6):1674-1688.
10. Huber M, *et al.* (2022) An RNA sponge controls quorum sensing dynamics and biofilm formation in Vibrio cholerae. *Nat Commun* 13(1):7585.
11. Culviner PH, Guegler CK, & Laub MT (2020) A Simple, Cost-Effective, and Robust Method for rRNA Depletion in RNA-Sequencing Studies. *mBio* 11(2).
12. Siemers M, Lippegaus A, & Papenfort K (2023) ChimericFragments: Computation, analysis, and visualization of global RNA networks. *bioRxiv* DOI: 10.1101/2023.12.21.572723.
13. Bar A, Argaman L, Altuvia Y, & Margalit H (2021) Prediction of Novel Bacterial Small RNAs From RIL-Seq RNA-RNA Interaction Data. *Front Microbiol* 12:635070.
14. Bruchmann S, *et al.* (2015) Deep transcriptome profiling of clinical Klebsiella pneumoniae isolates reveals strain and sequence type-specific adaptation. *Environ Microbiol* 17(11):4690-4710.
15. Hör J, Matera G, Vogel J, Gottesman S, & Storz G (2020) Trans-Acting Small RNAs and Their Effects on Gene Expression in Escherichia coli and Salmonella enterica. *EcoSal Plus* 9(1).
16. Iosub IA, *et al.* (2021) The mRNA derived MalH sRNA contributes to alternative carbon source utilization by tuning maltoporin expression in E. coli. *RNA Biol* 18(6):914-931.
17. Bailey TL & Elkan C (1994) Fitting a mixture model by expectation maximization to discover motifs in biopolymers. *Proc Int Conf Intell Syst Mol Biol* 2:28-36.
18. Thelin KH & Taylor RK (1996) Toxin-coregulated pilus, but not mannose-sensitive hemagglutinin, is required for colonization by Vibrio cholerae O1 El Tor biotype and O139 strains. *Infect Immun* 64(7):2853-2856.
19. Figueroa-Bossi N, Valentini M, Malleret L, & Bossi L (2009) Caught at its own game: regulatory small RNA inactivated by an inducible transcript mimicking its target. *Gene Dev* 23(17):2004-2015.
20. Hoyos M, Huber M, Forstner KU, & Papenfort K (2020) Gene autoregulation by 3' UTR-derived bacterial small RNAs. *Elife* 9.
